# BKM120 sensitizes BRCA-proficient triple negative breast cancer cells to olaparib through regulating FOXM1 and Exo1 expression

**DOI:** 10.1038/s41598-021-82990-y

**Published:** 2021-02-26

**Authors:** Yu Li, Yuantao Wang, Wanpeng Zhang, Xinchen Wang, Lu Chen, Shuping Wang

**Affiliations:** 1 State Key Laboratory of Natural Medicines and Jiangsu Key Laboratory of Drug Design and Optimization, Department of Medicinal Chemistry, China Pharmaceutical University, Nanjing, 211198 P. R. China; 2 School of Life Science and Technology, China Pharmaceutical University, Nanjing, 211198 P. R. China

**Keywords:** Cancer, Breast cancer

## Abstract

Poly (ADP-ribose) polymerase (PARP) inhibitors offer a significant clinical benefit for triple-negative breast cancers (TNBCs) with BRCA1/2 mutation. However, the narrow clinical indication limits the development of PARP inhibitors. Phosphoinositide 3-kinase (PI3K) inhibition sensitizes BRCA-proficient TNBC to PARP inhibition, which broadens the indication of PARP inhibitors. Previously researches have reported that PI3K inhibition induced the defect of homologous recombination (HR) mediated repair by downregulating the expression of BRCA1/2 and Rad51. However, the mechanism for their synergistic effects in the treatment of TNBC is still unclear. Herein, we focused on DNA damage, DNA single-strand breaks (SSBs) repair and DNA double-strand breaks (DSBs) repair three aspects to investigate the mechanism of dual PI3K and PARP inhibition in DNA damage response. We found that dual PI3K and PARP inhibition with BKM120 and olaparib significantly reduced the proliferation of BRCA-proficient TNBC cell lines MDA-MB-231 and MDA231-LM2. BKM120 increased cellular ROS to cause DNA oxidative damage. Olaparib resulted in concomitant gain of PARP1, forkhead box M1 (FOXM1) and Exonuclease 1 (Exo1) while inhibited the activity of PARP. BKM120 downregulated the expression of PARP1 and PARP2 to assist olaparib in blocking PARP mediated repair of DNA SSBs. Meanwhile, BKM120 inhibited the expression of BRAC1/2 and Rad51/52 to block HR mediated repair through the PI3K/Akt/NFκB/c-Myc signaling pathway and PI3K/Akt/ FOXM1/Exo1 signaling pathway. BKM120 induced HR deficiency expanded the application of olaparib to HR proficient TNBCs. Our findings proved that PI3K inhibition impaired the repair of both DNA SSBs and DNA DSBs. FOXM1 and Exo1 are novel therapeutic targets that serves important roles in DNA damage response.

## Introduction

Poly (ADP-ribose) polymerases (PARPs) paly key role in DNA damage response network^1^. Especially PARP1 and PARP2, which are necessary for the repair of single-strand breaks (SSBs) through the base excision repair (BER) pathway^1–3^. PARP inhibition blocks the repair of DNA SSBs and causes double-strand breaks (DSBs)^4^. DSBs will activate cell death if unrepaired or promote unwanted chromosome rearrangements and genome instability if miss-repaired^4,5^. Homologous recombination (HR) mediated repair is the most effective repair that uses a sister chromatid template for recombination^4–6^. Tumor-suppressor gene breast cancer 1 (BRCA1) and BRCA2 are essential components of HR mediated repair that recruit to damaged DNA for DNA DSBs repair^7,8^. Loss or mutation of BRCA1/2 results in deficiency of HR mediated DNA DSBs repair^8^.Targeting PARP for synthetic lethality is a successful strategy for cancers with BRCA1/2 mutation^9–12^. PARP inhibitors have been successful used in the treatment of a small subset of triple-negative breast cancers (TNBCs) with BRCA mutation^9,10^. However, these mutations are rare, BRAC-proficient TNBC patients still would not benefit from therapy with PARP inhibitors^9,10,13^. Meanwhile, the adaptive resistance of cancers to PARP inhibitor also restricts the therapy of PARP inhibitors^14,15^. Therefore, TNBC remains an incurable illness, and therapeutic options for TNBCs are limited and based on the use of multiple lines of chemotherapy. New therapy is need to broad the use of PARP inhibitors and reverse drug resistance in the treatment of TNBCs.

Aberrant activation of the phosphoinositide 3-kinase (PI3K) signaling pathway is closely related to the development of TNBCs^16^. In addition to regulating cellular processes including metabolism, growth and survival, PI3K and its downstream genes are also necessary for DNA repair^8,9,16^. PI3K signaling pathway regulates the expression, location and phosphorylation of BRCA1 to stabilize DNA DSBs repair in TNBCs^17–19^. Inhibition of the PI3K signaling pathway has been proven to block HR mediated DNA repair by downregulating the expression of BRCA1 and Rad51, and subsequent sensitized BRCA-proficient TNBC to PARP inhibitors^8,9,20–23^. As the key genes involved in the PI3K signaling pathway, extracellular signal-regulated kinase (ERK) and its downstream ETS1 transcription factor involve in the regulation of BRCA1/2 and Rad51 expression^8,9^. Except for the ERK/ETS1 signaling pathway, there is little research on the mechanism of the synergistic effects of dual PI3K and PARP inhibition in the treatment of TNBCs^8,9,20–23^. Clarify their synergistic mechanism is necessary to broaden the therapies of PARP inhibitors.

Nuclear factor-кB (NFκB) and c-Myc are the downstream targets of protein kinase B (Akt), which involve in HR mediated DNA repair^24–26^. Akt directly induces the activation of NFκB and overexpression of c-Myc, and activated NFκB upregulates the expression of c-Myc^24,25^. Overexpression of the oncogene c-Myc causes the expression and activation of DNA damage repair Exonuclease 1 (Exo1), which is a key player in DNA DSBs repair^26^. The transcription factors forkhead box O1 (FOXO1) and forkhead box O3 (FoxO3a) are also the direct downstream genes of Akt that function in HR mediated DNA repair^27–29^. Constitutive activation of PI3K/ Akt signaling pathway directly results in phosphorylation of FOXOs and their subsequent cytoplasmic sequestration and/or degradation via the ubiquitin–proteasome pathway^27,29^. Although the case for FOXOs as tumor suppressors has become more complicated because of their alternative roles in stress response, FOXO1 and FoxO3a still function as tumor suppressors in solid tumor^27,30,31^. FOXO3a inhibits DNA damage repair through multiple pathways including regulation the function of forkhead transcription factor forkhead box M1 (FOXM1)^32^. FOXM1 involves in DNA damage response by regulating genes which play key roles in HR mediated repair of DNA DSBs, such as Exo1, BRCA1/2, Rad51/52^26,32–36^. Therefore, we hypothesized that PI3K inhibition would result in HR impairment through regulating the expression and activity of these downstream genes of Akt.

Herein, we selected the BRCA-proficient TNBC cell line MDA-MB-231 to investigate the synergistic effects of dual PI3K and PARP1 inhibition with BKM120 and olaparib in the treatment of BRCA-proficient TNBCs. We investigated the function of BKM120 in DNA damage, DNA SSBs repair and DNA DSBs repair to explain how PI3K inhibition sensitizes BRCA-proficient TNBC cells to PARP inhibitor. We found that BKM120 and olaparib synergistically inhibited the proliferation of MDA-MB-231 cells at the ratio of 1:6. BKM120 sensitized MDA-MB-231 cells to olaparib through inducing DNA damage, blocking DNA SSBs repair by downregulating the expression of PARP1/2, and inhibiting HR mediated DNA DSBs repair through the PI3K/Akt/NFκB/c-Myc signaling pathway and PI3K/Akt/FOXM1/Exo1 signaling pathway. BKM120 induced HR impairment expands the indication of olaparib. FOXM1 and Exo1 are novel therapeutic targets that serve important roles in DNA damage response.

## Results

### BKM120 and olaparib synergistically inhibit the growth of BRCA-deficient and BRCA-proficient TNBC cells

**Figure 1:**
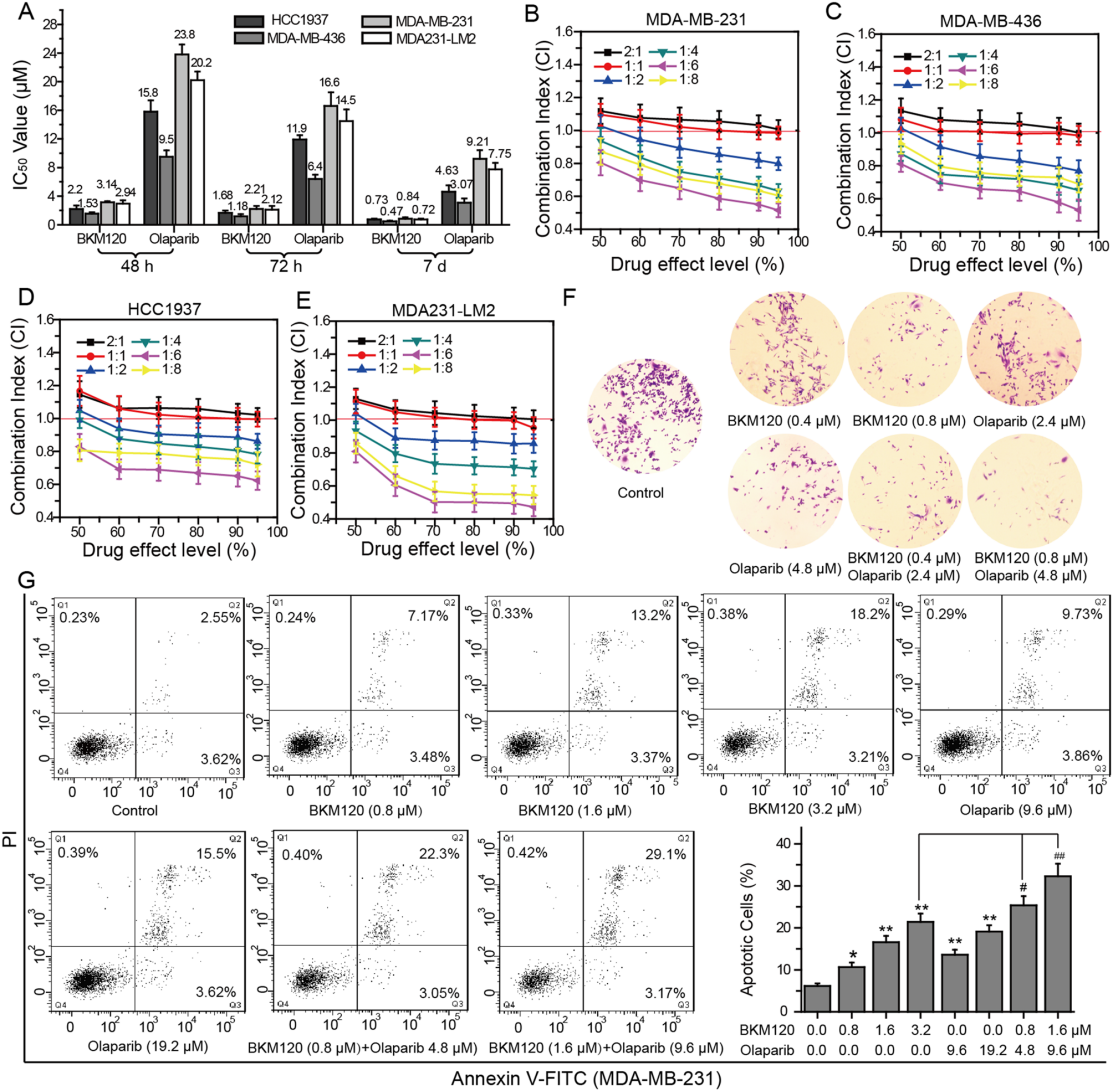
Synergistic effects of BKM120 and olaparib on the growth of TNBC cells. (**A**) IC_50_ values for the effects of BKM120 and olaparib on the viability of HCC1937, MDA-MB-436, MDA231-LM2 and MDA-MB-231 cells measured by MTT assay. CI curves for concurrent treatment with BKM120 and olaparib in (**B**) MDA-MB-231 cells, (**C**) MDA-MB-436 cells, (**D**) HCC1937 cells and (**E**) MDA231-LM2 cells. CI < 1.0 indicated a synergistic effect. The combination ratios of BKM120/olaparib are 2:1, 1:1, 1:2, 1:4, 1:6 and 1:8, respectively. (**F**) The combined effects of BKM120 and olaparib on the proliferation of MDA-MB-231 cells measured by colony-forming assay. (**G**) The combined effects of BKM120 and olaparib on the apoptosis of MDA-MB-231 cells. Apoptotic cells measured by Annexin V-FITC and PI double staining*.* Q_1_ and Q_4_ respectively represent the proportions of death cells and living cells, Q_2_ and Q_3_ were used to calculate apoptotic cells. Figures show a representative experiment of three independent experiments. *Error bars* represent SEM from the mean of three separate experiments. ^*^P < 0.05 and ^**^P < 0.01 compared to control. ^#^P < 0.05 and ^##^P < 0.01 compared to 3.2 μM BMK120-treated group.

PI3K inhibition has been proven to promote the sensibility of BRCA-proficient TNBC cells to PARP inhibitors^8,9,20–23^. Deficiency of BRCA1 causes HR impairment and induces TNBC cells sensitize to PARP inhibitors, but proficiency of BRCA1 dose not^24^. Thus, we selected BRCA1 deficient TNBC cell lines MDA-MB-436 and HCC1937, and BRCA-proficient TNBC cell lines MDA-MB-231 and high migration TNBC cell line MDA231-LM2 to determine the effects of dual PI3K and PARP1 inhibition on the proliferation of BRCA-deficient and BRCA-proficient TNBC cells^8,9^. The effects of BKM120, olaparib, or their combination on the proliferation of TNBC cells were determined by MTT assay (Fig. 1A and Supplementary Fig. 1). The effects of BKM120 and olaparib on the growth of TNBC cells increased with the increasing of treatment time. After 7 days of treatment, the therapeutic effects of olaparib dramatically increased. Compared with olaparib, the four TNBC cell lines were more sensitive to BKM120. Meanwhile, the inhibition of BKM120 on the proliferation of the four TNBC cells were no significant difference (Fig. 1A and Supplementary Fig. 1). The inhibitory effects of olaparib on the viability of MDA-MB-436 cells and HCC1937 cells were higher than its inhibitory effects on MDA-MB-231 cells and MDA231-LM2 cells (Fig. 1A and Supplementary Fig. 1). After 72 h of treatment with olaparib, the IC_50_ value for the BRCA-deficient TNBC cell line MDA-MB-436 was 6.4 ± 0.7 μM, but the IC_50_ value for the BRCA-proficient TNBC cell line MDA-MB-231 was 16.6 ± 2.1 μM (Fig. 1A). The combination effects of BKM120 and olaparib at fixed dose ratio (BKM120/olaparib are 2:1, 1:1, 1:2, 1:4, 1:6, and 1:8) on the growth of TNBC cells were investigated (Fig. 1B–E). Combing BKM120 and olaparib not only inhibited the growth of BRCA-deficient TNBC lines MDA-MB-236 and HCC1937, but also impaired the growth of BRCA-proficient TNBC lines MDA-MB-231 and MDA231-LM2 (Fig. 1B–E). After analyzed the CI curves, we found the dose ratio of BKM120/olaparib was in the range of 1:2 to 1:8, the combination of BKM120 and olaparib yielded synergistic effect (CI < 1.0) on the growth of TNBC cells. 1:6 was the best dose ratio for BKM120/olaparib to exert their synergistic effects (Fig. 1B–E). As shown in Table 1, combing 0.99 ± 0.05 μM BKM120 with 5.94 ± 0.30 μM olaparib caused 50% of cell death, which needed 2.21 ± 0.17 μM BKM120 alone or 16.6 ± 0.82 μM olaparib alone to achieve the same effects. The corresponding DRI values for BKM120 and olaparib are 2.23 ± 0.21 (DRI_BKM120_) and 2.79 ± 0.19 (DRI_olaparib_), respectively. The CI value is 0.80 ± 0.07, which proved that combing BKM120 with olaparib at ratio of 1:6 (BKM120/olaparib) synergistically inhibited the proliferation of MDA-MB-231 cells. Colony formation assay was performed to confirm the synergistic effects of BKM120 and olaparib on the proliferation of BRCA-proficient TNBC cell lines MDA-MB-231. The results showed that combing 0.8 μM BKM120 with 4.8 μM olaparib dramatically inhibited colony forming (Fig. 1F).

**Table 1:** DRI and CI values for the effects of combining BKM120 with olaparib on the proliferation of MDA-MB-231 cells.

Inhibition ratio	BKM120 (μM)	Olaparib (μM)	Ratio (1:6)		BKM120 (DRI)	Olaparib (DRI)	CI value
			BKM120 (μM)	Olaparib (μM)			
0.50	2.21 ± 0.17	16.6 ± 0.82	0.99 ± 0.05**	5.94 ± 0.30^##^	2.23 ± 0.21	2.79 ± 0.19	0.80 ± 0.09
0.60	3.46 ± 0.18	25.1 ± 2.02	1.32 ± 0.06**	7.92 ± 0.36^##^	2.57 ± 0.18	3.24 ± 0.30	0.70 ± 0.08
0.70	5.03 ± 0.27	36.5 ± 1.96	1.70 ± 0.07**	10.2 ± 0.42^##^	2.87 ± 0.18	3.34 ± 0.22	0.65 ± 0.06
0.80	6.44 ± 0.71	40.2 ± 1.61	1.92 ± 0.11**	11.5 ± 0.66^##^	3.35 ± 0.42	3.49 ± 0.24	0.58 ± 0.08
0.90	8.78 ± 0.83	49.7 ± 1.64	2.36 ± 0.13**	14.2 ± 0.78^##^	3.81 ± 0.41	3.51 ± 0.23	0.55 ± 0.07
0.95	10.9 ± 0.97	55.3 ± 2.31	2.62 ± 0.12**	15.7 ± 0.72^##^	4.16 ± 0.42	3.52 ± 0.22	0.52 ± 0.06

*P < 0.05 and **P < 0.01 compared to BKM120 alone treated group, ^#^P < 0.05 and ^##^P < 0.01 compared to olaparib alone treated group.

Subsequently, we investigated the effects of BKM120 and olaparib on the apoptosis of MDA-MB-231 cells (Fig. 1G). Q_2_ and Q_3_ represent the percentage of Annexin V-FITC^(+)^/PI^(+)^ and Annexin V-FITC^(+)^/PI^(−)^ cells, respectively. After 72 h of treatment, 3.2 μM BKM120 and 19.2 μM olaparib obviously promoted the apoptosis of MDA-MB-231 cells. Compared with control, 3.2 μM BKM120 increased the percentage of apoptotic cells from 6.17 ± 0.6 to 21.41 ± 2.6%, and 19.2 μM olaparib increased the percentage from 6.17 ± 0.6 to 19.12 ± 1.6%. Combining BKM120 with olaparib synergistically increased cell apoptosis. Compared with 3.2 μM BKM120 alone treated group, the combination of 0.8 μM BKM120 with 4.8 μM olaparib increased the percentage of apoptotic cells from 21.41 ± 2.6 to 25.35 ± 2.3%, and the combination of 1.6 μM BKM120 with 9.6 μM olaparib dramatically increased the percentage from 21.41 ± 2.6 to 32.27 ± 3.3% (Fig. 1G). The data suggest that dual PI3K and PARP1 inhibition with BKM120 and olaparib synergistically inhibits the growth of BRCA-proficient TNBC cells.

### BKM120 and olaparib synergistically induce DNA damage in MDA-MB-231 cells

**Figure 2:**
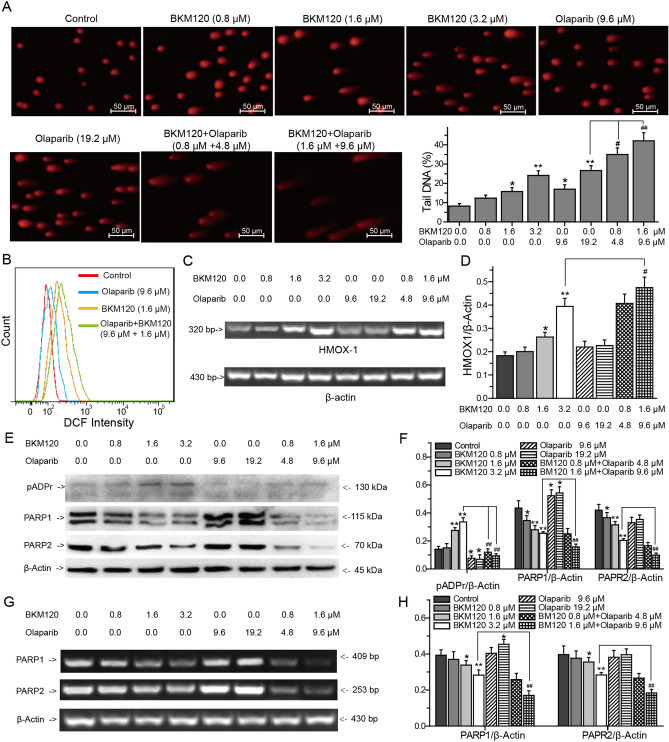
Effects of BKM120 and olaparib on DNA damage in MDA-MB-231 cells. (**A**) DNA damage was assessed by alkaline comet assay. The tail DNA percentage of comet assay at indicated concentration of BKM120, olaparib, and their combination. (**B**) Effects of drugs on cellular ROS analyzed by flow cytometry. (**C**) RT-PCR analysis of the expression of HMOX-1 induced by drugs. (**D**) Percentage of relative intensity obtained from the corresponding RT-PCR. (**E**) Effects of drugs on the expressions of pADPr, PARP1 and PARP2 analyzed by western blots. (**F**) Relative intensity expression obtained from the corresponding western blots. (**G**) Effects of drugs on the expressions of PARP1 and PARP2 in MDA-MB-231 cells analyzed by RT-PCR. (**H**) Relative intensity expression obtained from the corresponding RT-PCR. *Error bars* represent SEM from the mean of three separate experiments. ^*^P < 0.05 and ^**^P < 0.01 compared to control, ^#^P < 0.05 and ^##^P < 0.01 compared to 3.2 μM BMK120-treated group.

The effects of BKM120, olaparib or their combination on DNA damage were determined by comet assay (Fig. 2A). After 72 h of treatment, both 3.2 μM BKM120 and 19.2 μM olaparib induced DNA damage in the BRCA-deficient MDA-MB-231 cells. Compared with 19.2 μM olaparib treated group, combining BKM120 with olaparib synergistically increased DNA damage (Fig. 2A). Compared with 19.2 μM olaparib treated group, 0.8 μM BKM120 and 4.8 μM olaparib increased the tail DNA percentage of MDA-MB-231 cells from 26.12 ± 2.5% to 34.97 ± 3.4%, and 1.6 μM BKM120 and 9.6 μM olaparib dramatically increased the percentage from 26.12 ± 2.5 to 42.12 ± 4.3% (Fig. 2A).

### BKM120 directly induces DNA oxidative damage to synergize with olaparib

Accumulation of cellular ROS cause oxidative damage to DNA. The results of Comet assay showed that both BKM120 and olaparib induced the damage of DNA in MDA-MB-231 cells. Therefore, we detected the effects of BKM120 and olaparib on cellular ROS to investigate whether BKM120 and olaparib can directly induce endogenous oxidative damage to DNA. The effects of BKM120 and olaparib on the generation of cellular ROS were firstly determined with DCFH-DA fluorescence probe by flow cytometry. After 72 h of treatment, 1.6 μM BKM120 increased the percentage of DCF-positive cells, indicating an increase of cellular ROS (Fig. 2B)^37^. Compared with control, the effect of 9.6 μM olaparib on the accumulation of cellular ROS was not obvious. However, the combination of 1.6 μM BKM120 with 9.6 μM olaparib prominently increased cellular ROS (Fig. 2B). Collectively, these results indicated that 1.6 μM BKM120 directly induced oxidative damage to DNA, but olaparib did not. To confirm the conclusion, we also analyzed the effects of BKM120, olaparib and their combination on the expression of heme oxygenase-1 (HMOX1), a key oxidative stress response enzyme that is upregulated in the presence of elevated ROS, by RT-PCR (Fig. 2C)^38^. The results of RT-PCR showed that 1.6 and 3.2 μM BKM120 promoted the expression of HMOX1 (Fig. 2C,D). Compared with 3.2 μM BKM120 alone treated group, combing BKM120 with olaparib significantly increased the expression of HMOX1 (Fig. 2C,D). Notably, BKM120 resulted in the accumulation of cellular ROS to induce DNA damage.

### BKM120 directly inhibits the expression of PARP1 and PARP2 to synergize with olaparib in the repair of DNA SSBs

PARP1 and PARP2 are involved in the repair of DNA SSBs. Poly(ADP-ripose) (pADPr) proteins is a product of PARP activation, which reflects to the activation of PARP^8^. Olaparib promotes the death of BRCA-deficient cells through inhibiting the activity of PARP1/2^8,9^. Thus, we analyzed the effects of BKM120, olaparib and their combination on the expression of pADPr, PARP1 and PARP2 by RT-PCR and western-blots to expose their functions in the repair of DNA SSBs. The results of western-blots showed that 3.2 μM BKM120 resulted in concomitant gain of pADPr, olaparib blocked the generation of pADPr by inhibiting the activity of PARP1 (Fig. 2E,F). Adding of olaparib impaired the accumulation of pADPr induced by BKM120 (Fig. 2E,F). RT-PCR and western-blots analysis showed an interesting discovery that olaparib upregulated the expression of PARP1 while it inhibited the activity of PARP1 (Fig. 2E–G). BKM120 downregulated the expression of PARP1 and PARP2 to inhibit the repair of DNA SSBs. Compared with BKM120 alone or olaparib alone treated group, the combination of BKM120 with olaparib synergistically enhanced the downregulation of PARP1 and PARP2 (Fig. 2F,H).

### BKM120 downregulates BRCA1/2 and Rad51/52 to synergize with olaparib in HR mediated repair of DNA DSBs

**Figure 3:**
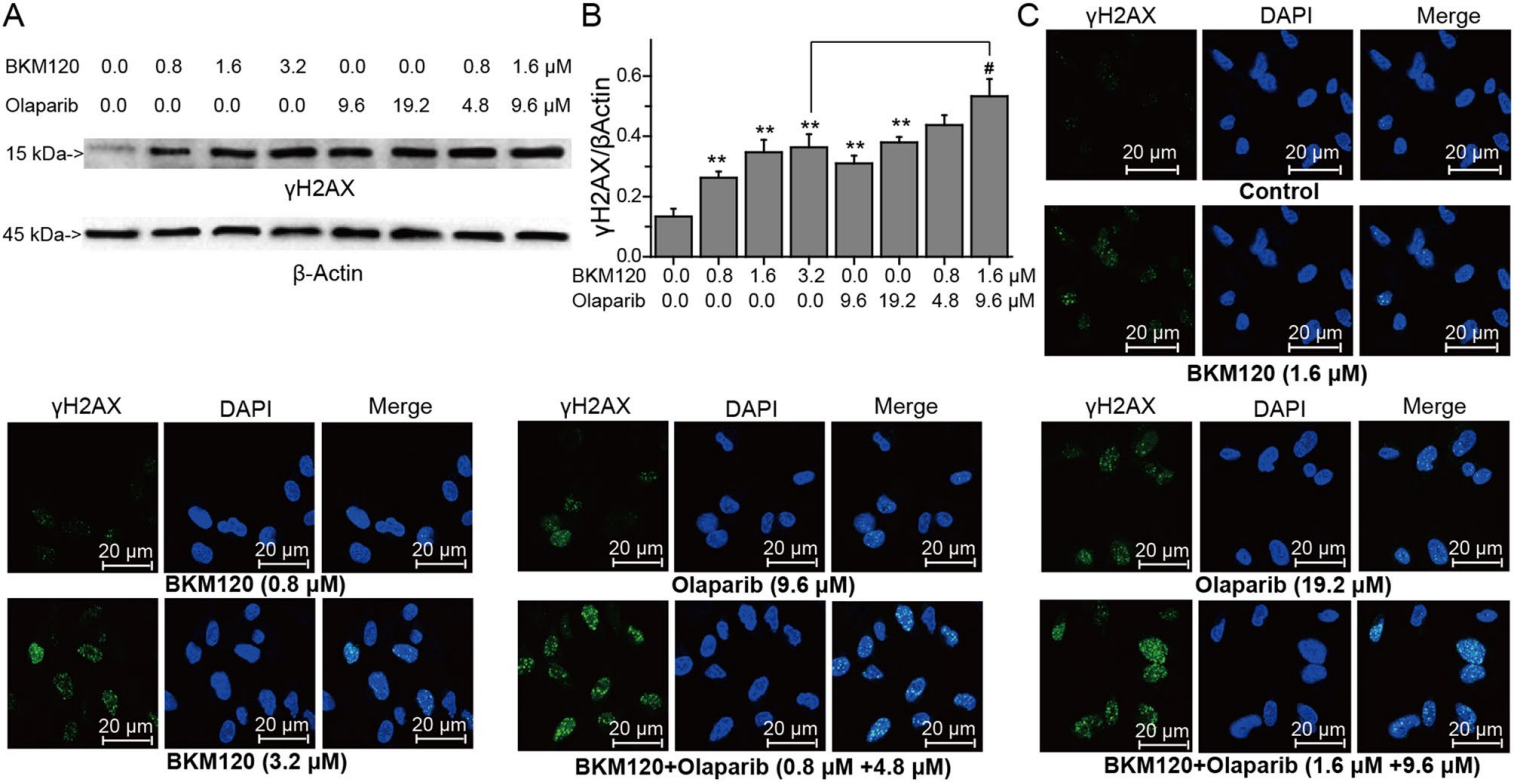
Synergistic effects of BKM120 and Olaparib on the expression of γh2AX. (**A**) Effects of BKM120, olaparib, and their combination on the expressions of γh2AX analyzed by western blots. (**B**) Relative intensity expression obtained from Western blots. (**C**) Effects of drugs on the expression of γh2AX analyzed by Immunofluorescence. Green represents the MDA-MB-231 cells stained with anti-γh2AX antibody, Blue represents the nuclei stained with DAPI. *Error bars* represent SEM from the mean of three separate experiments. ^*^P < 0.05 and ^**^P < 0.01 compared to control, ^#^P < 0.05 and ^##^P < 0.01 compared to 3.2 μM BMK120-treated group.

γH2AX is a protein that localizes to damaged DNA and recruits DNA repair effectors to these sites^9,39^. The accumulation of γH2AX reflects the degree of damaged DNA, especially DNA with DSBs^39^. The results of western-blots and immunofluorescence showed that BKM120 obviously increased the accumulation of γh2AX in nucleus (Fig. 3). Compared with 3.2 μM BKM120 alone treated group, the combination of BKM120 and olaparib significantly promoted the accumulation of γH2AX to enhancing DSBs of DNA (Fig. 3A,B).

**Figure 4:**
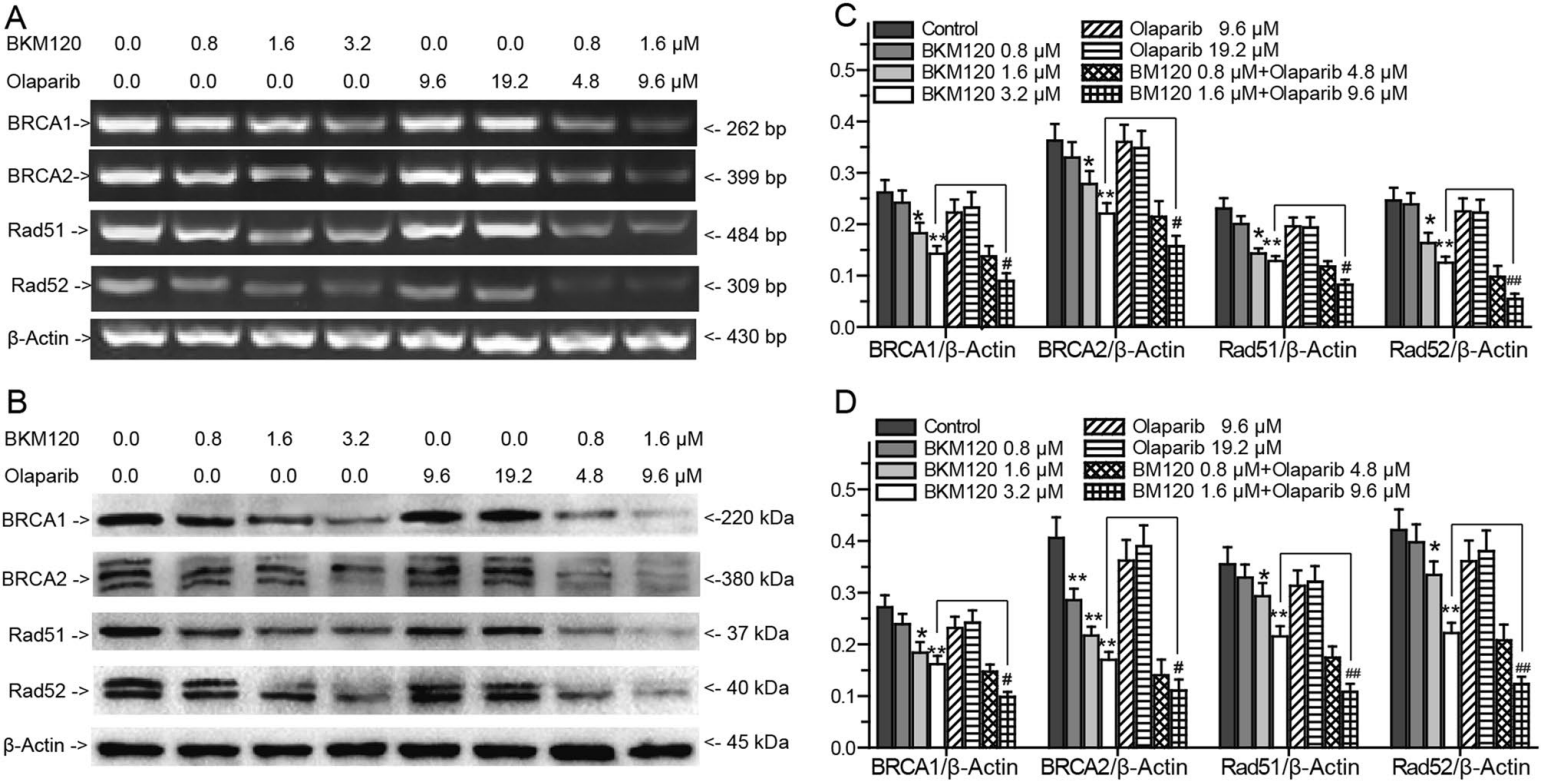
Effects of BKM120 and olaparib on the repair factors of DNA DSBs. Effects of BKM120, olaparib, and a combination of BKM120 with olaparib on the expressions of BRCA1, BRCA2, Rad51 and Rad52 in MDA-MB-231 cells analyzed by (**A**) RT-PCR and (**B**) western blots. Relative intensity expression obtained from the corresponding (**C**) RT-PCR and (**D**) western blots. *Error bars* represent SEM from the mean of three separate experiments. ^*^P < 0.05 and ^**^P < 0.01 compared to control, ^#^P < 0.05 and ^##^P < 0.01 compared to 3.2 μM BMK120-treated group.

BRCA1, BRCA2, Rad51 and Rad52 are crucial for HR mediated repair of DNA DSBs^8,9,40^. To prove whether BKM120 causes the HR impairment to increase the sensitivity of MDA-MB-231 cells to olaparib, we investigated the effects of BKM120, olaparib and their combination on the expression of BRCA1, BRCA2, Rad51 and Rad52 by PCR and western-blots (Fig. 4). Olaparib did not regulate the expression of BRCA1, BRCA2, Rad51 and Rad52 (Fig. 4). BKM120 caused the decreasing of BRCA1, BRCA2, Rad51 and Rad52 expression (Fig. 4). Compared with 3.2 μM BKM120 alone treated group, the combination of 1.6 μM BKM120 with 9.6 μM olaparib prominently inhibited the expression of these HR repair factors (Fig. 4C,D). Overall, BKM120 blocked HR mediated repair of DNA DSBs by impairing the expression of BRCA1, BRCA2, Rad51 and Rad52 to sensitize MDA-MB-231 cells to olaparib.

### PI3K/Akt/NFκB/c-Myc signaling pathway involves in BKM120 induced HR impairment

**Figure 5:**
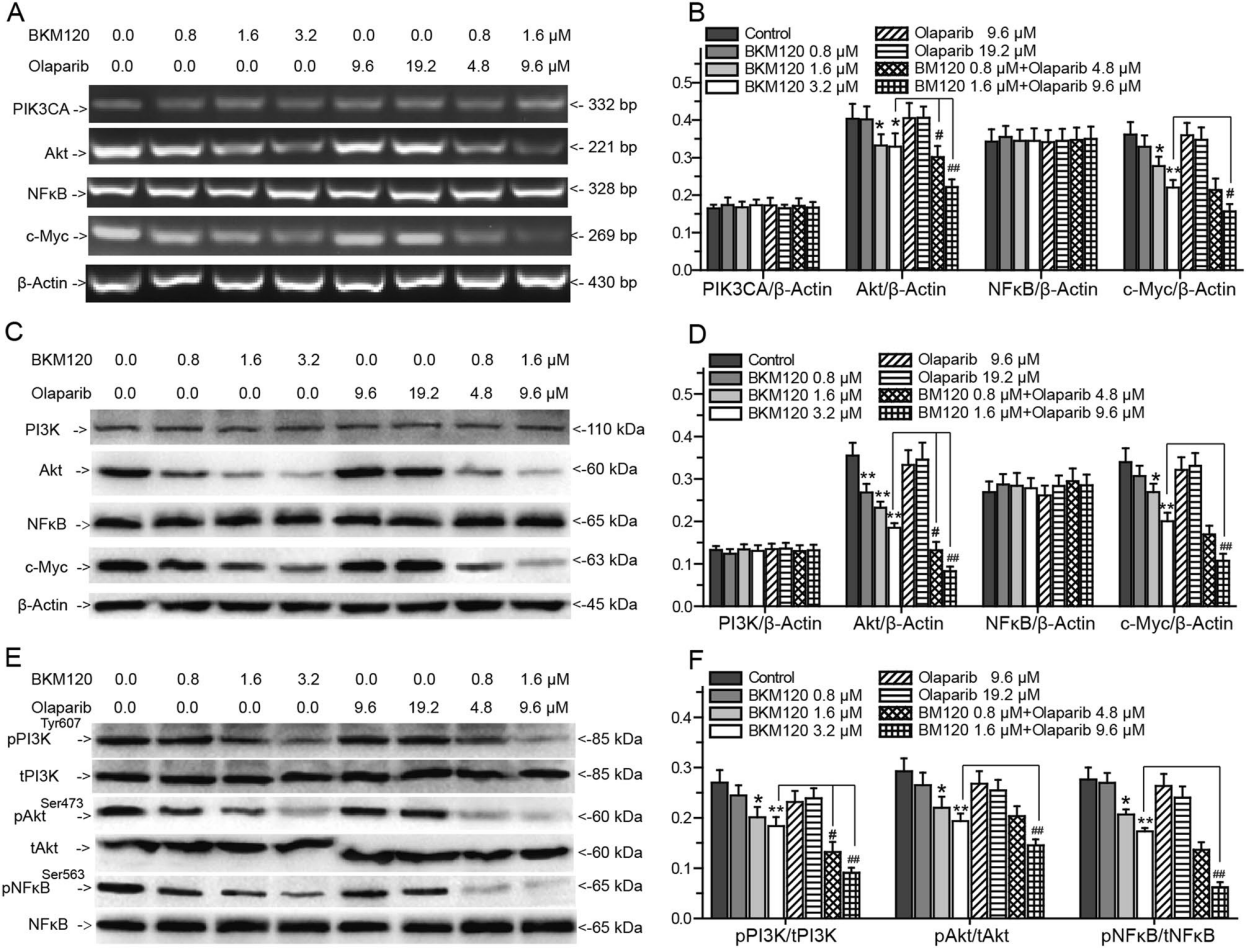
Effects of BKM120 and olaparib on the PI3K/Akt/NFκB/c-Myc signaling pathway. Effects of BKM120, olaparib, and a combination of BKM120 and olaparib on the expressions of PI3K, Akt, NFκB and c-Myc in MDA-MB-231 cells analyzed by (**A**) RT-PCR and western blots (**C**). (**E**) Effects of drugs on the phosphorylation of PI3K, Akt, and NFκB analyzed by western blots. Relative intensity expression obtained from the corresponding (**B**) RT-PCR and (**E,F**) western blots. *Error bars* represent SEM from the mean of three separate experiments. ^*^P < 0.05 and ^**^P < 0.01 compared to control, ^#^P < 0.05 and ^##^P < 0.01 compared to 3.2 μM BMK120-treated group.

NFκB and c-Myc, the downstream targets of Akt, play key roles in HR mediated repair and cell apoptosis^24–26^. We hypothesized that the PI3K/Akt/NFκB/c-Myc signaling pathway might be involved in BKM120 induced HR impairment. Thus, we treated MDA-MB-231 cells with BKM120, olaparib or their combination and determined the expression and activation of genes involved in the PI3K/Akt/NFκB/c-Myc signaling pathway by RT-PCR and western-blots (Fig. 5). As the inhibitor of PI3K, BKM120 did not regulated the expression of PI3K catalytic p110 subunit, but blocked the phosphorylation of PI3K regulatory p85 subunit (Fig. 5). Phosphorylation of the PI3K regulatory p85 subunit results in resistance against PI3K inhibitor^41^. Impairment of the phosphorylation of p85 inhibits the activation of PI3K/Akt signaling pathways^42^. Thus, BKM120 impaired the phosphorylation of PI3K to inhibit the function of PI3K. Meanwhile, BKM120 downregulated the expression of Akt and pAkt^Ser473^ to inhibit its expression and activity (Fig. 5). BKM120 did not regulate the expression of NFκB, but inhibited the expression of pNFκB^Ser563^ to inhibit its activity (Fig. 5). Moreover, the expression of c-Myc was also impaired by BKM120 (Fig. 5A–D). However, olaparib did not show significant regulation in the expression and phosphorylation of above proteins (Fig. 5). BKM120 and olaparib synergistically regulated these targets involved in the PI3K/Akt/NFκB/c-Myc signaling pathway. Compared with 3.2 μM BKM120 alone treated group, combing 1.6 μM BKM120 with 9.6 μM olaparib obviously blocked the expression of Akt and c-Myc, and downregulated the expression of pPI3K^tyr607^, pAkt^Ser473^, pNFκB^Ser563^ to regulate their activity (Fig. 5).

### PI3K/Akt/FOXM1/Exo1 signaling pathway plays key role in BKM120 induced impairment

**Figure 6:**
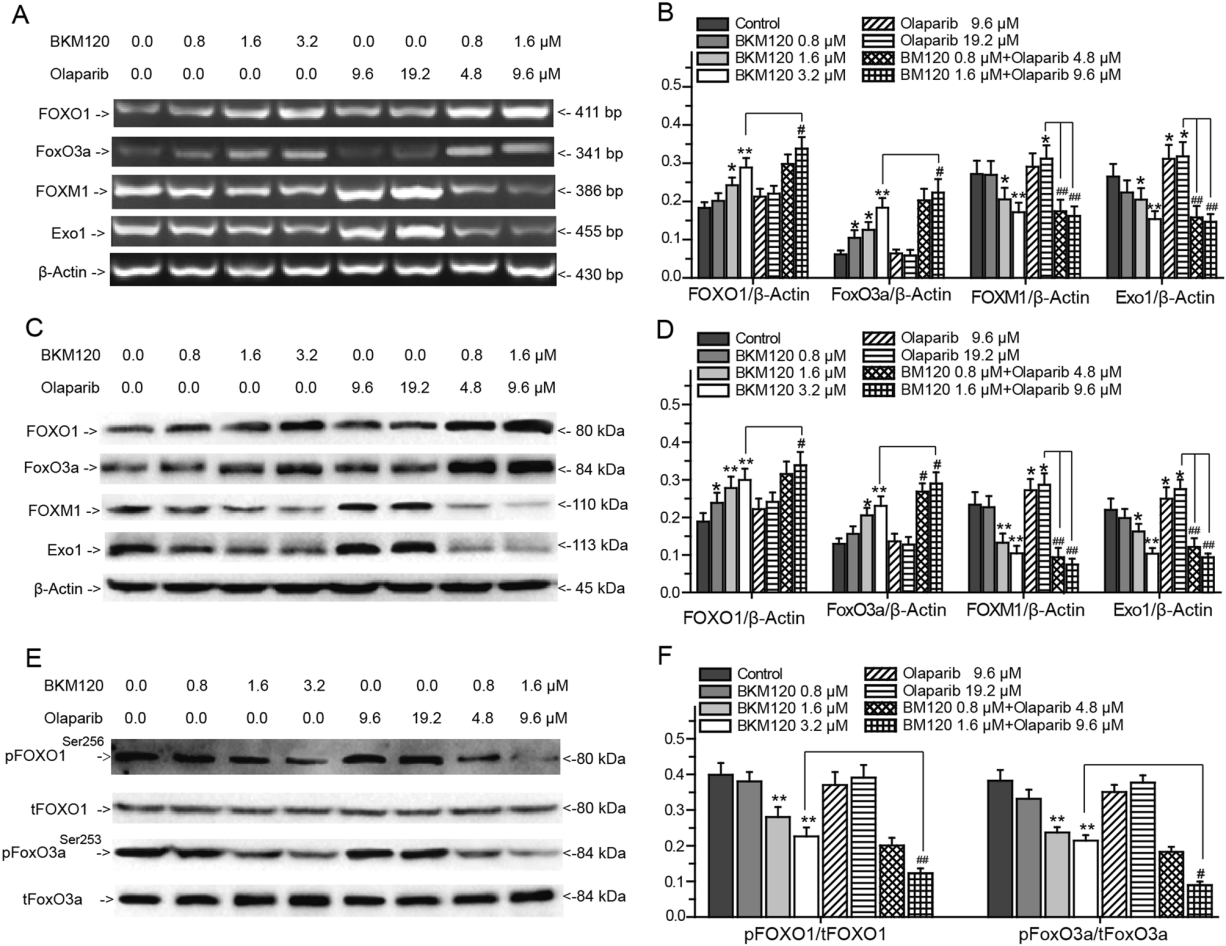
Effects of BKM120 and olaparib on the PI3K/Akt /FOXM1/Exo1 signaling pathway. Effects of BKM120, olaparib, and a combination of BKM120 with olaparib on the expressions of FOXO1, FoxO3a, FOXM1 and Exo1 in MDA-MB-231 cells analyzed by (**A**) RT-PCR and (**C**) western blots. (**E**) Effects of drugs on the phosphorylation of FOXO1 and FoxO3a analyzed by western blots. Relative intensity expression obtained from the corresponding (**B**) RT-PCR and (**E,F**) western blots. *Error bars* represent SEM from the mean of three separate experiments. ^*^P < 0.05 and ^**^P < 0.01 compared to control, ^#^P < 0.05 and ^##^P < 0.01 compared to 19.2 μΜ olaparib-treated group.

As the directly downstream targets of Akt, the FOXOs family members, especially FOXO1 and FoxO3a also participate in DNA damage response^32–36^. We evaluated the effects of BKM120, olaparib, or their combination on the expression and activation of FOXO1, FoxO3a and their downstream genes FOXM1 and Exo1, to clarify the synergistic effects of BKM120 and olaparib on the HR mediated repair of DNA DSBs (Fig. 6). After 72 h of treatment, olaparib increased the expression of pFoxO3a^Ser253^, FOXM1, and Exo1, but did not regulate the expression of FOXO1, pFOXO1^Ser256^, and FoxO3a (Fig. 6). The above results showed that olaparib promoted the HR mediated repair while inhibiting the repair of DNA SSBs. On the contrary, BKM120 dramatically upregulated the expression of FOXO1 and FoxO3a, inhibited the phosphorylation of FOXO1 and FoxO3a, and downregulated the expression of FOXM1 and Exo1 (Fig. 6). Meanwhile, combing BKM120 with olaparib synergistically regulated the FoxO3a/FOXM1/Exo1 signaling pathway to inhibit HR mediated repair (Fig. 6). Compared with 3.2 μM BKM120 alone treated group, combining BKM120 with olaparib obviously increased the expression of FOXO1 and FoxO3a, and impaired the expression of pFOXO1^Ser256^ and pFoxO3a^Ser253^ (Fig. 6). Combining BKM120 with olaparib did not obviously inhibit the expression of FOXM1 and Exo1. Adding of BKM120 dramatically blocked the expression of FOXM1 and Exo1 which increased by olaparib (Fig. 6A–D).

### FOXM1 and Exo1 directly downregulate the expression of BRCA1/2 and Rad51/52 to cause DNA damage

**Figure 7:**
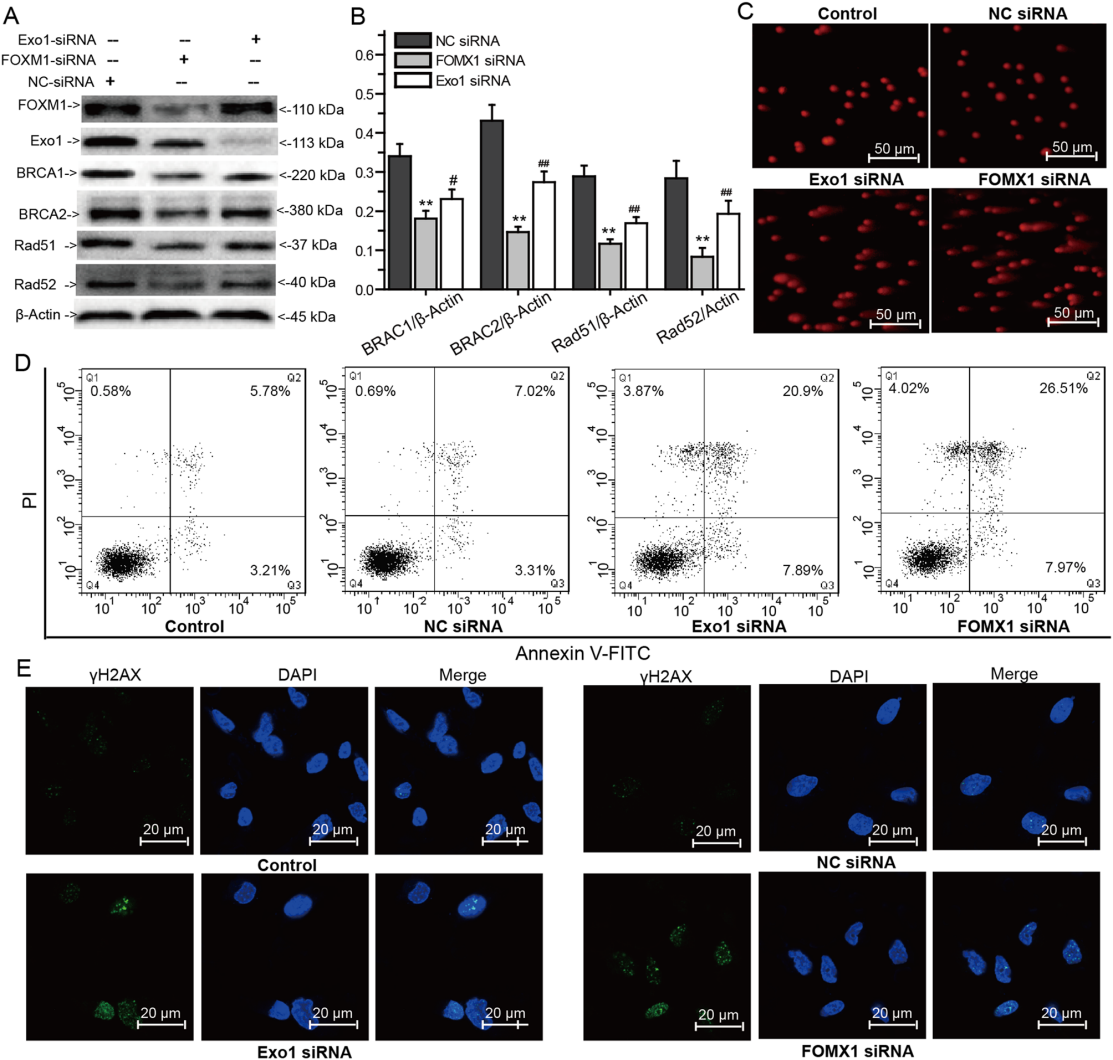
The roles of FOXM1 and Exo1 in the repair of DNA DSBs. (**A**) The expression of BRCA1, BRCA2, Rad51 and Rad52 after silence the expression of FOXM1 and Exo1 analyzed by western blots. (**B**) Relative intensity expression obtained from the corresponding western blots. (**C**) Comet assay analysis of DNA damage after silence the expression of FOXM1 and Exo1. (**D**) Apoptosis of MDA-MB-231 cells measured by Annexin V-FITC and PI double staining after silence the expression of FOXM1 and Exo1. (**E**) Immunofluorescence analysis of γh2AX after silence the expression of FOXM1 and Exo1. Error bars represent SEM from the mean of three separate experiments. ^*^P < 0.05 and ^**^P < 0.01 represent FOXM1 siRNA group compared to NC siRNA group, ^#^P < 0.05 and ^##^P < 0.01 represent Exo1 siRNA group compared to NC siRNA group.

To confirm whether FOXM1 and Exo1 involved in HR mediated repair of DNA DSBs, we knocked down the expression of FOXM1 and Exo1 in MDA-MB-231 cells using FOXM1 siRNA and Exo1 siRNA, with the scrambled negative siRNA as control (NC siRNA). FOXM1 siRNA inhibited the expression of FOXM1 and Exo1, but Exo1 siRNA only inhibited the expression of Exo1 expression (Fig. 7A,B). Compared with the NC siRNA treated group, silence the expression of FOXM1 or Exo1 significantly inhibited the expression of BRCA1, BRCA2, Rad51 and Rad52 (Fig. 7A,B). According to comet assay and apoptosis assay, knockdown of FOXM1 or Exo1 induced DNA damage and cell apoptosis (Fig. 7C,D). The expression of γH2AX was also observed in the knockdown experiments, whereas the silence of FOXM1 or Exo1 increased γH2AX expression, indicating the key roles of FOXM1 and Exo1 in HR mediated repair of DNA DSBs (Fig. 7E).

## Discussion

Targeting PARP for synethetic lethality is an effective strategy for BRCA-deficient TNBCs and ovarian cancers^8–11^. Olaparib and other PARP inhibitors have been successfully used in the treatment of TNBCs^20–22^. However, BRCA mutations are rare. Recovery of HR mediated DNA repair may result in adaptive resistance of cancers against PARP1 inhibitors^13–15^. PI3K signaling pathway has been proven to maintain HR steady state. PI3K inhibitors (LY294002, BKM120 and etc.) have been applied for treating triple negative breast cancer^17–23^. Additionally, many research works have showed that PI3K inhibition sensitized BRCA-proficient TNBCs to PARP inhibition, which expands the therapy of PARP inhibitors^8,9^. Inhibition of PI3K expression and activity has been proven to impair the expression of BRCA1/2 and Rad51 through ERK signaling pathway^8,9^. However, many genes involves in HR mediated repair of damaged DNA^24–33,40,43^. Investigating the function of BKM120 and olaparib in DNA damage response, and finding key regulatory genes of DNA repair are benefit to promote targeted therapy for TNBCs. Therefore, we selected PARP1 inhibitor olaparib and PI3K inhibitor BKM120 to investigate their synergistic effects on DNA damage response.

Herein, we investigated the synergistic effects of BKM120 and olaparib on DNA damage, the repair of DNA SSBs, and HR mediated repair of DNA DSBs. Combining BKM120 with olaparib not only inhibited the growth of BRCA deficient TNBC cell lines MDA-MB-436 and HCC1937 but also inhibited the proliferation of BRCA proficient TNBC cell lines MDA-MB-231 and MDA231-LM2. The results confirmed the conclusion that inhibition of PI3K promoted the sensitivity of BRCA proficient TNBC cells to PARP inhibitors^8,9,20–23^. BKM120, olaparib and their combination caused the accumulation of damaged DNA. BKM120 alone or combined with olaparib resulted in the accumulation of cellular ROS to induce oxidative damage of DNA, but olaparib did not. BKM120 directly induced DNA damage to promote the death of TNBC cells.

PARP1 and PARP2 are important factors in the repair of DNA SSBs. As the inhibitor of PARP, olaparib inhibited the activity of PARP1 and PARP2 to block PARP regulated repair of DNA SSBs. Meanwhile, olaparib caused compensated gain of PARP1 after 72 h of treatment. PARP1 overexpression results in adaptive resistance of breast cancer to olaparib^44^. Thus, olaparib caused resistance of TNBCs to itself by promoting the expression of PARP1. BKM120 resulted in decreased expression of PARP1 and PARP2, and concomitant gain of PAR protein, a maker of PARP activation^8,9^. The accumulation of PAR positively refers to the activation of PARP^8,9^. Therefore, BKM120 increased the activity of PARP while inhibiting the expression of PARP. Interestingly, combing BKM120 with olaparib impaired the expression and activity of PARP1 and PARP2. BKM120 reversed olaparib-induced PARP1 expression. Olaparib impaired BKM120 caused PAR gaining. BKM120 synergized with olaparib to induce the accumulation of damaged DNA by blocking PARP mediated repair of DNA SSBs.

γH2AX can localize to damaged DNA and recruit DNA repair factors to start DNA repair, especially HR mediated repair of DSBs^39^. As the mark of DNA DSBs, the accumulation of γH2AX reflects an increasing of DNA DSBs^39^. BRCA1, BRCA2, Rad51 and Rad52 are essential components of HR that are recruited to damaged DNA for repair^8,9,40^. As the inhibitor of PI3K, BKM120 caused the accumulation of γH2AX to enhance DNA damage, and induced the decreased expression of BRCA1, BRCA2, Rad51, and Rad52 to inhibit HR mediated repair of DNA DSBs. Although olaparib did not regulate the expression of above genes, BKM120 induced impairment of HR sensitized BRCA proficient MDA-MB-231 cells to olaparib. PI3K inhibition impairs HR mediated repair to sensitize BRCA proficient TNBC cells to PARP1 inhibitors, but the corresponding mechanism is unclear^8,9^. Akt is the directly downstream gene of PI3K that has many downstream targets that involved in HR mediated repair of damaged DNA^24–26,32–35^. Except for the ERK signaling pathway, we hypothesis that other pathways might be involved in BKM120 induced HR impairment. NFκB and c-Myc are the downstream genes that involved in HR repair^8,24–26^. The c-Myc, which is also regulated by NFκB, regulates the expression of Exo1 to participate in DNA damage repair^21,22^. As the inhibitor of PI3K, BKM120 binds to the catalytic p110 subunit to inhibit the activity of PI3K^8.9^. We found that BKM120 also impaired the phosphorylation of the regulatory p85 subunit to regulate the PI3K/Akt signaling pathway^41,42^. Really, BKM120 inhibited the phosphorylation of Akt and NFκB, downregulated Akt and c-Myc expression, but olaparib did not regulate these genes. Combing BKM120 with olaparib synergistically inhibited the activation of Akt and NFκB, and impaired the expression of c-Myc to induce HR impairment.

The forkhead box family proteins FOXO1, FoxO3a and FOXM1 are also proved to involve in HR mediated repair^28,32–35^. FOXO1 and FoxO3a are the directly targets of Akt, and FOXM1 are the downstream factor of FoxO3a^29^. Among these forkhead box family genes, FOXM1 is very important for damaged DNA repair and described as an “emerging master regulator of DNA damage response”^33,45^. FOXM1 always functions by regulating the expression of Exo1, which is an evolutionarily nuclease that participates in DNA DSBs repair^33–36^. We found that olaparib increased the expression of FOXM1 and Exo1 to promote HR mediated repair of DNA DSBs. On the contrary, BKM120 decreased FOXM1 and Exo1 expression to block HR mediated repair. BKM120 reversed olaparib-induced negative feedback in HR mediated repair by reducing olaparib-induced accumulation of FOXM1 and Exo1. Further mechanism study showed that BKM120 promoted the expression and inhibited phosphorylation of FOXO1 and FoxO3a. The phosphorylation of FOXO1 and FoxO3a causes they transferred from nucleus to cytoplasm, which inhibits their transcription function^28–32^. Thus, BKM120 reduced olaparib-induced expression of FOXM1 and Exo1 through promoting the accumulation of FOXO1 and FoxO3a in nucleus. BRCA1/2 and Rad51 are the target genes of FOXM1 and Exo1^34,35^. Rad52 is also an essential gene that involves in HR mediated repair^40^. Except for BRCA1/2 and Rad51, our results showed that BKM120 and olaparib inhibited the expression of Rad52. We silenced the expression of FOXM1 and Exo1, and found that the expression of BRCA1, BRCA2, Rad51 and Rad52 were impaired. Inhibition of FOXM1 and Exo1 expression blocked HR repair to cause DNA damage and cell death.

**Figure 8:**
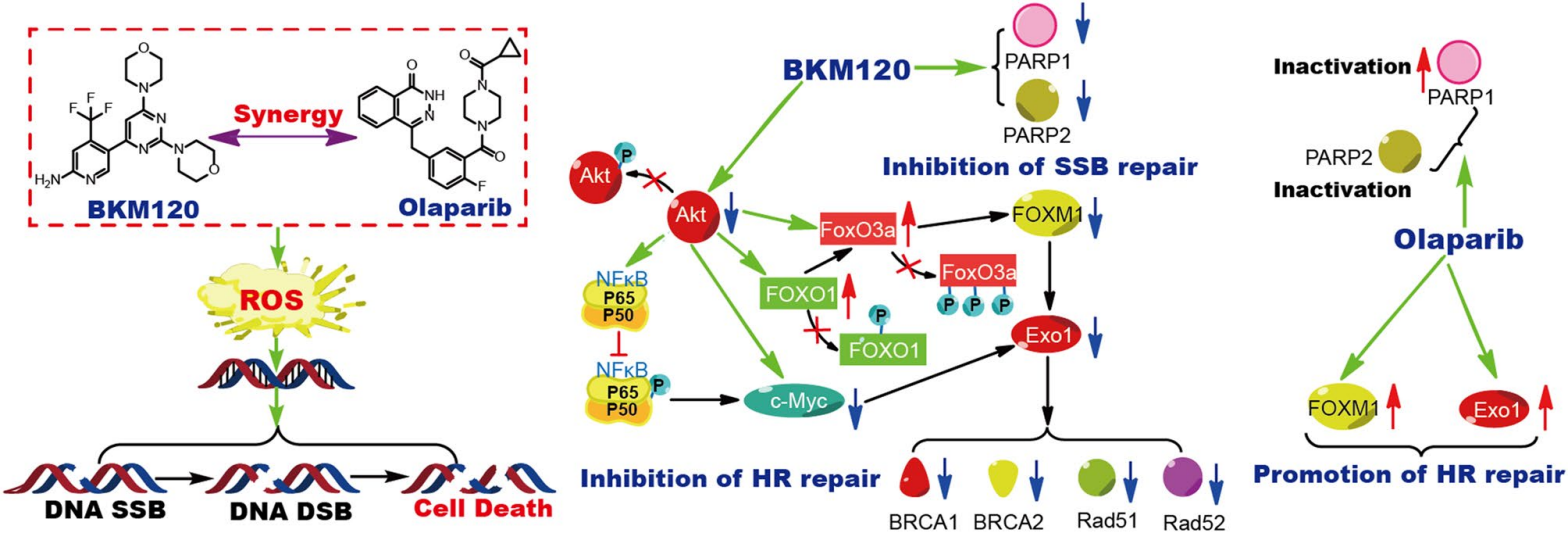
Mechanism for the synergistic effects of BKM120 and olaparib on the death of MDA-MB-231 cells. BKM120 directly induces DNA damage through increasing cellular ROS. Meanwhile, BKM120 downregulates the expression of PARP1 and PARP2 to synergy the inhibition of olaparib in the repair of DNA single damage. Moreover, BKM120 inhibits the HR repair of DNA double damage to sensitize cells to olaparib through regulation the PI3K/Akt/NFĸB/c-Myc pathway and PI3K/Akt/ /FOXM1/Exo1 pathway.

In summary, we clarified the mechanism for the synergistic effect of BKM120 and olaparib on the growth of BRCA proficient TNBC cells. Olaparib upregulated the expression of PARP1, FOXM1 and Exo1, while inhibiting the activity of PARP1. The accumulation of PARP1, FOXM1 and Exo1 promoted the repair of DNA SSBs and DNA DSBs, and caused adaptive resistance of TNBC cells to olaparib. BKM120 promoted cell death through direct inducing oxidative damage of DNA and blocking the repair of DNA SSBs and DNA DSBs. Moreover, BKM120 sensitized BRCA proficient cell line MDA-MB-231 to olaparib through regulating DNA damage response. As shown in Fig. 8, combing BKM120 with olaparib directly induced oxidative damage of DNA, inhibited PARP1/2 expression and the activity of PARP1. BKM120 inhibited olaparib-induced FOXM1 and Exo1 expression by regulating the PI3K/Akt/NFκB/c-Myc signaling pathway and PI3K/Akt/FOXM1/Exo1 signaling pathway. When BKM120 impaired the expression of FOXM1 and Exo1, then blocked the expression of BRCA1/2 and Rad51/52 to induce HR impairment (Fig. 8). However, there are many effective inhibitors of PI3K and PARP, our work only selected BKM120 and olaparib for mechanism research. Thus, the mechanism of dual PI3K and PARP inhibition in DNA damage response needs more PI3K and PARP inhibitors to confirm.

## Materials and methods

### Cell culture and cell viability assay

BRCA proficient TNBC cell line MDA-MB-231, and the BRCA deficient TNBC cell lines MDA-MB-436 and HCC1937 were purchased from Cell Resources Center of Shanghai Academy of life sciences (Shanghai, China). The high mobility BRCA proficient TNBC cell line MDA231-LM2 was brought from China Center for Type Culture Collection (Wuhan, China). MDA-MB-231 cells and MDA231-LM2 cells were cultured in Leibovitz’s L-15 (KeyGEN Biotech, China) with 10% fetal bovine serum (FBS). HCC1937 cells were cultured in RPMI-1640 (KeyGEN Biotech, China) with 10% FBS. MDA-MB-436 cells were cultured in Leibovitz’s L-15 (KeyGEN Biotech, China) with 10% FBS, 10 mcg/ml insulin and 16 mcg/ml glutathione. All cells were incubated at 37 °C under a 5% CO_2_ atmosphere. Cell viability was measured by MTT cell proliferation assay kit according to the manufacturer’s protocols (KeyGEN Biotech, China). In brief, Cells were seeded at 2 × 10^4^ cells/ml in a 96 well culture plate and then exposed on various concentrations of BKM120 and olaparib for 48 h and 72 h. Untreated cells served as control.

### Drug combination assay

According to the IC_50_ values of BKM120 and olaparib for cell viability, the combination ratios of BKM120/olaparib were selected as 2:1, 1:1, 1:2, 1:4, 1:6 and 1:8, respectively. After cells were treated with BKM120 and olaparib at different combination ratios for 72 h, the proliferation of MDA-MB-231 cells, MDA231-LM2 cells, MDA-MB-436 cells and HCC1937 were measured by MTT assay. Untreated cells served as control. The dose reduction index (DRI) and combination index (CI) values were calculated by CompuSyn software using equation: CI = C_A,X_/IC_X,A_ + C_B,X_/IC_X,B_, CI = 1/DRI_A_ + 1/DRI_B_, DRI_A_ = IC_X,A_/C_A,X_, DRI_B_ = IC_X,B_/C_B,X_^44^_._ Herein, C_A,X_ and C_B,X_ respectively represent the concentrations of BKM120 and olaparib used in combination to achieve X% drug effect. The IC_X, A_ and IC_X, B_ are the concentrations for single drugs (BKM120 or olaparib) that achieve the same effect. The value of DRI is proportional to the combination effect of drugs. Synergy is defined as CI < 1, additivity is defined as CI = 1 and antagonism is defined as CI > 1^46^.

### Colony formation assay

MDA-MB-231 cells were seeded in 6-well plates at a concentration of 500 cells/well. After seeding of 12 h, the cells were exposed on various concentrations of BKM120, olaparib or their combination for another 3 days before changing with fresh media. The medium was changed every 2–3 days to form colonies. After 14 days of treatment, the colonies were fixed with 90% ethanol for 10 min, stained with 0.1% crystal violet for 10 min, and imaged using upright biological microscope (Olympus BX53, Tokyo, Japan). Each experiment was repeated three times. Untreated cells served as control^39^.

### Alkaline comet assay

Alkaline comet assay was performed using the comet assay kit according to the manufacturer’s protocol (KeyGEN Biotech, China). After the MDA-MB-231 cells were treated with different concentrations of BKM120, olaparib or their combination for 72 h, the cells were digested from the dishes and washed twice by Ca^2+^-free PBS. Untreated cells served as control. The cells (1 × 10^4^/ml) mixed with low melting point agarose at a ratio of 1:10 (v/v) were layered onto the Slide. The in gel cells were lysed by the lysis buffer at 4 °C for 2 h and then were unwound by alkaline unwinding solution for another 30 min at room temperature. Following an electrophoresis at 21 V for 30 min, the cells were stained with PI and were observed with the inverted biological microscope (Olympus BX53, Tokyo, Japan). Five images were randomly captured per slide. Nuclei were analyzed randomly 50 each from 3 slides per treatment and expressed as percent of tail DNA. The percentage of tail DNA [tail DNA (%)] was selected among the comet parameters as it gives us a clear indication of the extent of DNA damage induced by the test chemical^39^.

### Analysis of apoptosis and cellular ROS by flow cytometry

MDA-MB-231 cells were analyzed by MACSQuant flow cytometry (Cologne, Germany) to determine cell apoptosis and cellular ROS. Cell apoptosis was analyzed by Annexin V-FITC and propidium iodide (PI) apoptosis detection kit (KeyGEN Biotech). After 72 h of treatment, the cells were collected and stained with Annexin V-FITC and PI. Cellular reactive oxygen species (ROS) were determined by the 2′,7′-dichlorodihydrofluorescein diacetate (DCFH-DA) fluorescent probe. The contents of cellular 2′,7′-dichlorofluorescein (DCF) were positively correlated with ROS. After 72 h of treatment, MDA-MB-231 cells were collected and incubated in Leibovitz’s L-15 medium containing 10 μM DCFH-DA at 37 °C for 30 min. The treated cells were washed twice with PBS to remove excess probes for further analysis. The data of cell apoptosis and Cellular ROS were analyzed by FlowJo 7.6^33,34^.

### Immunofluorescence assay

MDA-MB-231 cells were treated as described in the text. The cells were collected and fixed with 4% formaldehyde, permeabilized with 0.1% (v/v) Triton X-100 in PBS and blocked with 2% (w/v) BSA in PBS for 1 h. After blocking, cells were incubated with the anti-pHistone 2AX (γH2AX) (ser139) mouse antibody with an Alexa-Flou-488-conjugation for 12 h. Cellular DNA was counterstained with 4,6-diamidino-2-phenylindole (DAPI; molecular probes). Fluorescence signals were detected using a Carl Zeiss LSM700 Laser confocal microscope (Jena, Germany). Five fields per sample were quantified with a threshold of 10 foci or more being considered positive^8^.

### RT-PCR analysis

Total RNA was extracted from MDA-MB-231 cells by RNAiso Plus (Takara, Dalian, China). Isolated total RNA (2 μg) was used to perform the reverse transcription with the PrimeScript RT reagent kit (Takara, Dalian, China) according to the manufacturer’s protocols. The transcribed cDNA (2 μL) was used for PCR amplification with specific primers in Table 2. Thirty cycles were carried under the following conditions: 30 s denaturation at 94 °C, 30 s annealing at T_m_ of each primers, and 30 s extension at 72 °C. The PCR products were separated on 1% agarose gel electrophoresis gel containing ethidium bromide^34,40^. The separated bands were imaged on a BioRad Chemidoc XRS^+^ System (Hercules, CA, USA).

**Table 2:** Primer sequences for the apoptosis factors in RT-PCR.

Name	Sense (5′-3′)	Antisense (5′-3′)
HMOX1	CCTAAACTTCAGAGGGGGCG	ATGGCTCAAAAACCACCCCA
PARP1	CGCCTGTCCAAGAAGATGGT	AAGGCACTTGCTGCTTGTTG
PARP2	AGCAAGATGAATCTGTGAAGGC	AGGCTGTGCTGTCCCATTTT
BRCA1	AATTGCGGGAGGAAAATGGG	CTTCACCACAGAAGCACCAC
BRCA2	GAAGCGTGAGGGGACAGATT	GATTGGTACAGCGGCAGAGT
Rad51	CTACTGGCTCCAAAGAGCTTGA	ACCACTGCTACACCAAACTCATC
Rad52	CCGAGGCGCAGGTCAAC	ATCCACATTCTGCTGCGTGA
c-Myc	TTTGCACTGGAACTTACAACACC	CCTCCTCGTCGCAGTAGAAAT
NFкB	ACTACGAGGGACCAGCCAAGA	CGCAGCCGCACTATACTCA
PIK3CA	CCACGCAGGACTGAGTAACA	CCAAGCACCGAACAGCAAAA
Akt	CTCTTTCCAGACCCACGACC	TAATGTGCCCGTCCTTGTCC
FOXO1	ACAGCCCTGGATCACAGTTT	TTTGGTAGTTTGGGCTGGGT
FoxO3a	GAGAGCTCCCCGGACAAAC	CTGTCGTCAGCTGATTCGGG
FOXM1	GAGCAGCGACAGGTTAAGGT	TGTGATTCCAAGTGCTCGGG
Exo1	CGGGCCAACAATACCTTCCT	TCTTCTGACAGCTCTGCACT
β-Actin	GACCTGACTGACTACCTC	TCTTCATTGTGCTGGGTGC

### Western-blot analysis

Total proteins in MDA-MB-231 cells were extracted by ice-cold RIPA cell lysis buffer (Beyotime, Shanghai, China). Total proteins (30 μg) of each sample were equally loaded onto SDS-PAGE electrophoresed gels and transferred to a PVDF membrane (Millipore, Billerica, MA, USA). After blocking with 5% (w/v) skim milk at room temperature for 1 h, the membranes were incubated with primary antibody in Supplementary Methods, followed by incubation with secondary antibodies (1:2000) for 1 h at room temperature. Proteins on the PVDF membrane were visualized using chemiluminescent HRP substrate (Millipore, Billerica, MA, USA). The intensities of the bands were corrected with respect to that of β-Actin. All the experiments were repeated three or more times^34,40^.

### siRNA transfections

FOXM1 siRNA, Exo1 siRNA and NC siRNA were brought from Santa Cruz biotechnology. FOXM1 siRNA was a mixture of three target specific siRNA that used to inhibit the expression of FOXM1. Exo1 siRNA was also a mixture of three Exo1 specific siRNA that used to block Exo1 expression. MDA-MB-231 cells were transfected with 100 nM control, FOXM1 or Exo1 siRNA using Lipofectamine 2000 (Cell signaling technology, Boston, MA, USA) according to the manufacturer’s instructions. Following 48 h of transfection, cells were harvested, and FOXM1, Exo1 knockdown were confirmed by immunoblotting the cell lysates with the anti-FOXM1 antibody and anti-Exo1-antibody respectively. After 48 h of transfection, the cells were collected to perform the experiments of western-blot, Comet assay, Immunofluorescence and apoptosis assay according to the above methods.

### Statistical analysis

Data were analyzed using SPSS 19.0. Results were expressed as means ± standard error of the mean. Differences between treatment regimens were analyzed by two tailed Student’s *t*-test. P < 0.05 was considered as statistical significance.

## Supplementary Information

Supplementary Information 1.

## Data Availability

All data are contained within the manuscript.
