# Supplementary material for: BKM120 sensitizes BRCA-proficient triple negative breast cancer cells to olaparib through regulating FOXM1 and Exo1 expression
Source: Sci Rep. 2021 Feb 26;11:4774. doi: 10.1038/s41598-021-82990-y (PMC7910492; doi:10.1038/s41598-021-82990-y)
Supplement: Supplementary file 1 — Supplementary Information 1. [file 41598_2021_82990_MOESM1_ESM.doc]

**Online Supplement**

**BKM120 sensitizes BRCA-proficient triple negative breast cancer cells to olaparib through regulating FOXM1 and Exo1 expression**

Yu Li^1^, Yuantao Wang^2^, Wanpeng Zhang^2^, Xinchen Wang^1^, Lu Chen^1^, Shuping Wang^*,1^

*^1^State Key Laboratory of Natural Medicines and Jiangsu Key Laboratory of Drug Design and Optimization, Department of Medicinal Chemistry, China Pharmaceutical University, Nanjing 211198, P. R. China*

*^2^School of Life Science and Technology, China Pharmaceutical University, Nanjing 211198, P. R. China*

1. **Materials and Methods**

**Reagents**

Anti-Rad52 (F-7) mouse mAb, anti-c-Myc (9E10) mouse mAb, anti-pADPr (10H) mouse mAb, anti-Exo1 (266) mouse mAb, anti-FOXM1 (A-11) mouse mAb,, human FOXM1 siRNA, human Exo1 siRNA and control siRNA were obtained from Santa cruz biotechnology (Santa Cruz, CA, USA). Anti-BRCA1 rabbit pAb and anti-BRCA2 rabbit pAb were bought from Proteintech Group (Chicago, IL, USA). Anti-Rad51 (D4B10) rabbit mAb, anti-FoxO3a (D19A7) rabbit mAb, anti-phospho-FoxO3a (Ser253) (D18H8) rabbit mAb, anti-NF-kappaB p65 (C22B4) rabbit mAb, anti-phospho-NF-kappaB, anti-AKT rabbit mAb, anti-PI3K p85α (6G10) mouse mAb, anti-PI3K p110α rabbit mAb, anti-FoxO1 (C29H4) rabbit mAb, anti-phospho-FoxO1 (Ser256) rabbit mAb, anti-β-Actin (8H10D10) mouse mAb, anti-mouse igG-HRP-linked antibody, anti-rabbit igG-HRP-linked antibody, and Lipofectamine 2000 transfection reagent were purchased from Cell signaling technology (Boston, MA, USA). Anti-AKT1 (phospho S473) rabbit mAb, anti-PI3K P85α (phospho Y607) rabbit pAb, anti-PARP1 rabbit mAb, and anti-PARP2 rabbit mAb were obtained from Abcam (Cambridge, England). Alexa Flour 488 Anti-Human/mouse phospho-Histone H2AX (Ser139) mouse mAb was bought from Affymetrix ebioscience (Santiago,CA, USA). BKM120, Olaparib, bovine serum albumin (BSA), insulin and glutathione were obtained from Sigma-Aldrich (St. Louis, MO, USA). Annexin V-FITC and propidium iodide (PI) apoptosis detection kit, MTT cell proliferation assay kit, 2’,7’-dichlorodihydrofluorescein diacetate (DCFH-DA), DAPI, comet assay kit, and crystal violet were bought from KeyGEN Biotech (Nanjing, China). The stock solution of BKM120 and Olaparib was prepared by transferring 10 mg to the DMSO at a concentration of 20 mM. Aliquots of the stock solutions were stored at -20 °C. All other chemicals used were analytical grade without purification.

1. **Supplemental Figures**


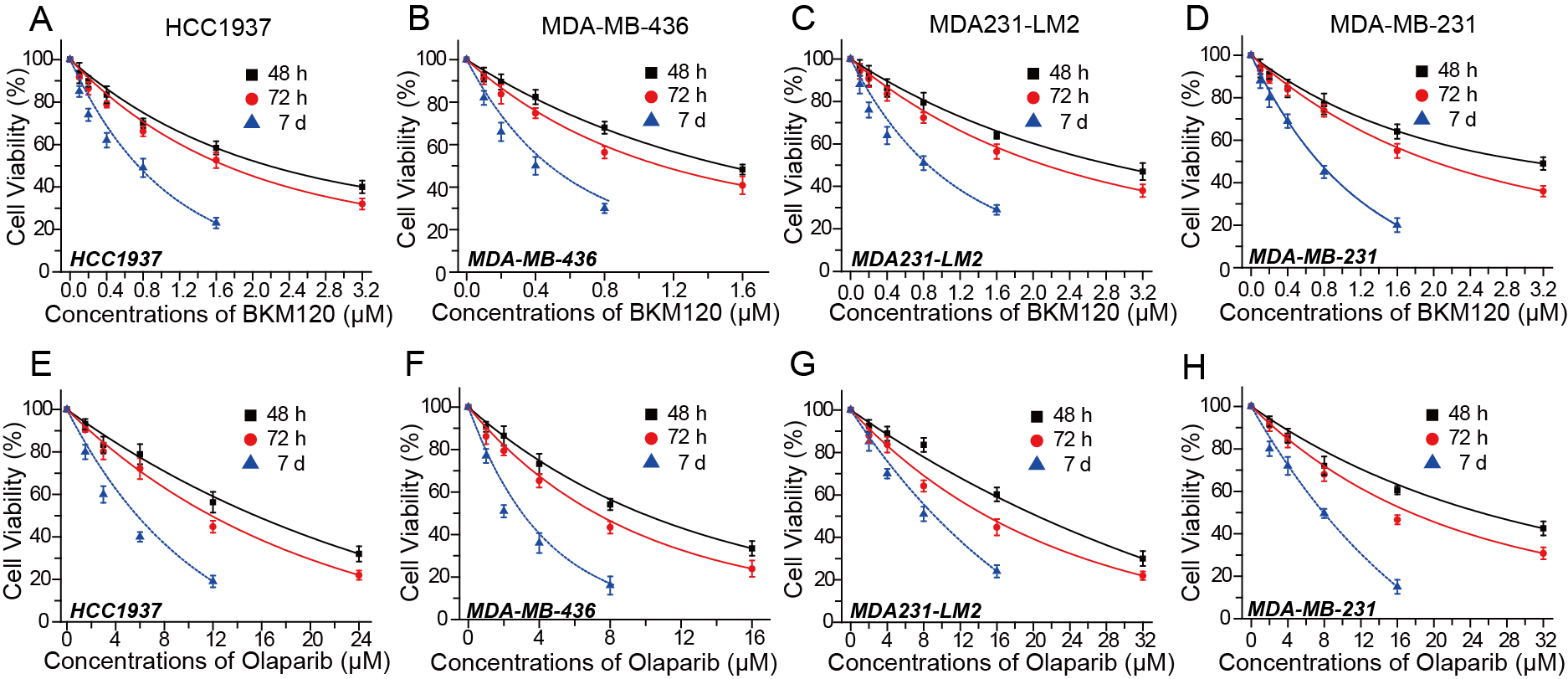


**Figure S1. The effects of BKM120 or olaparib on the proliferation of TNBC cells.** The effects of BKM120 on the proliferation of (A) HCC1937, (B) MDA-MB-436, (C) MDA231-LM2, (D) MDA-MB-231 cells. The effects of olaparib on the proliferation of (E) HCC1937, (F) MDA-MB-436, (G) MDA231-LM2, (H) MDA-MB-231 cells.


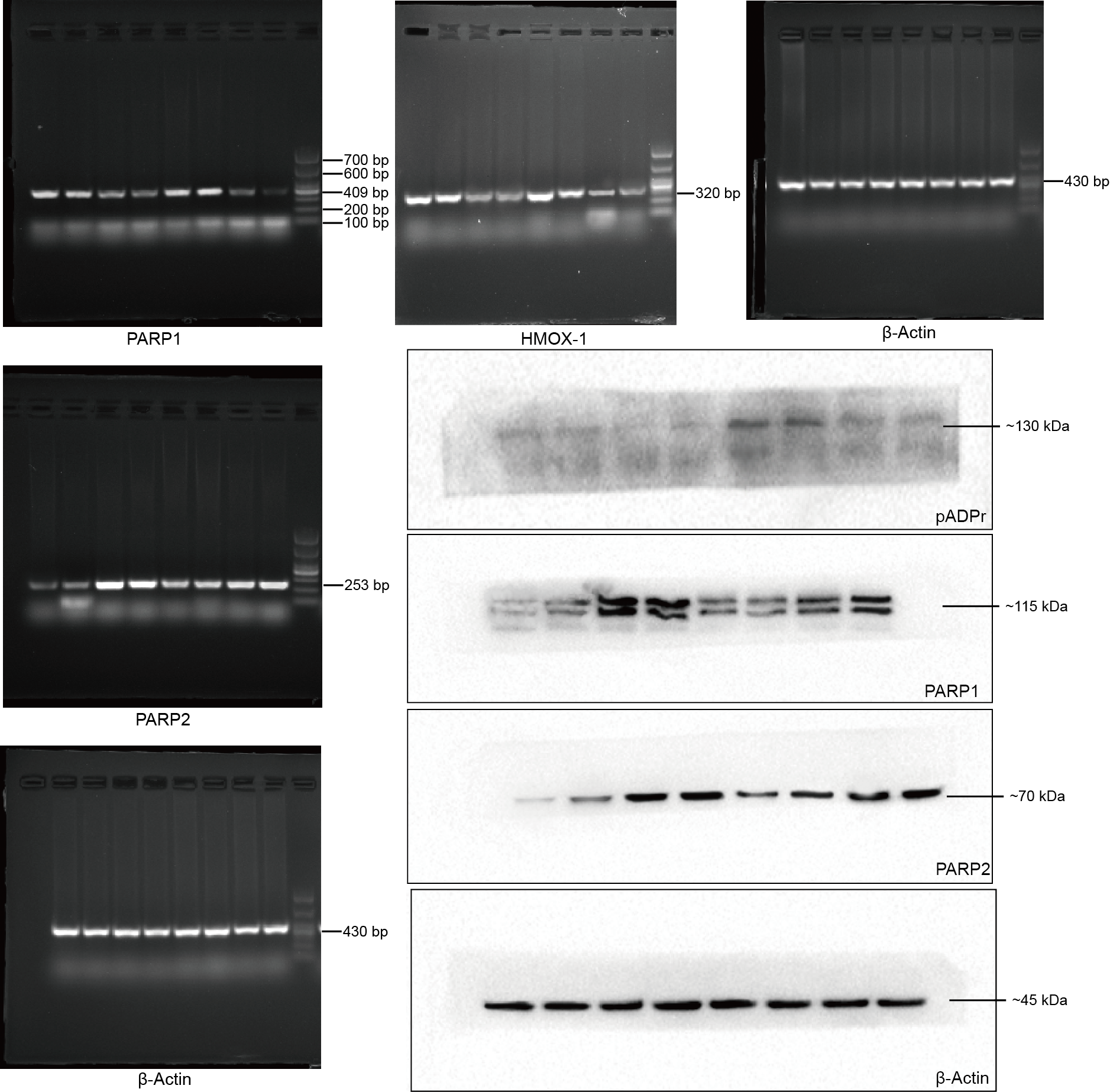


**Figure S2. Full length images of pADPr, PARP1, PARP2 and β-Actin.** The dilution ratios of all primary antibodies are 1:1000. The marker of DNA ladder from top to down are 700 bp, 600 bp, 500 bp, 400 bp, 300 bp, 200 bp and 100 bp, respectively.

**
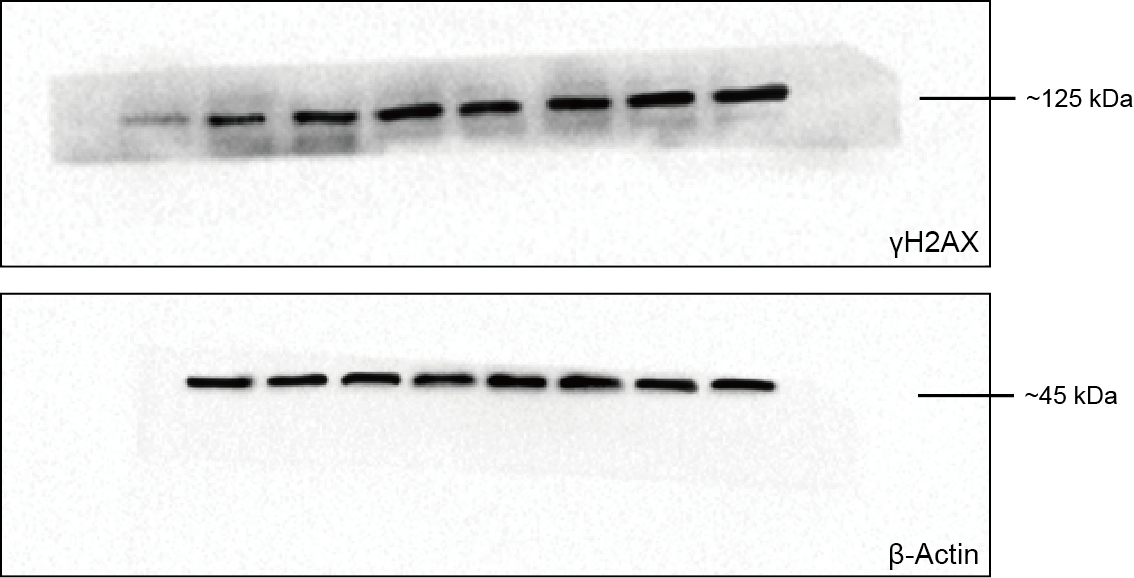
**

**Figure S3. Full length images of γH2AX and β-Actin.** The dilution ratios of all primary antibodies are 1:1000.

**
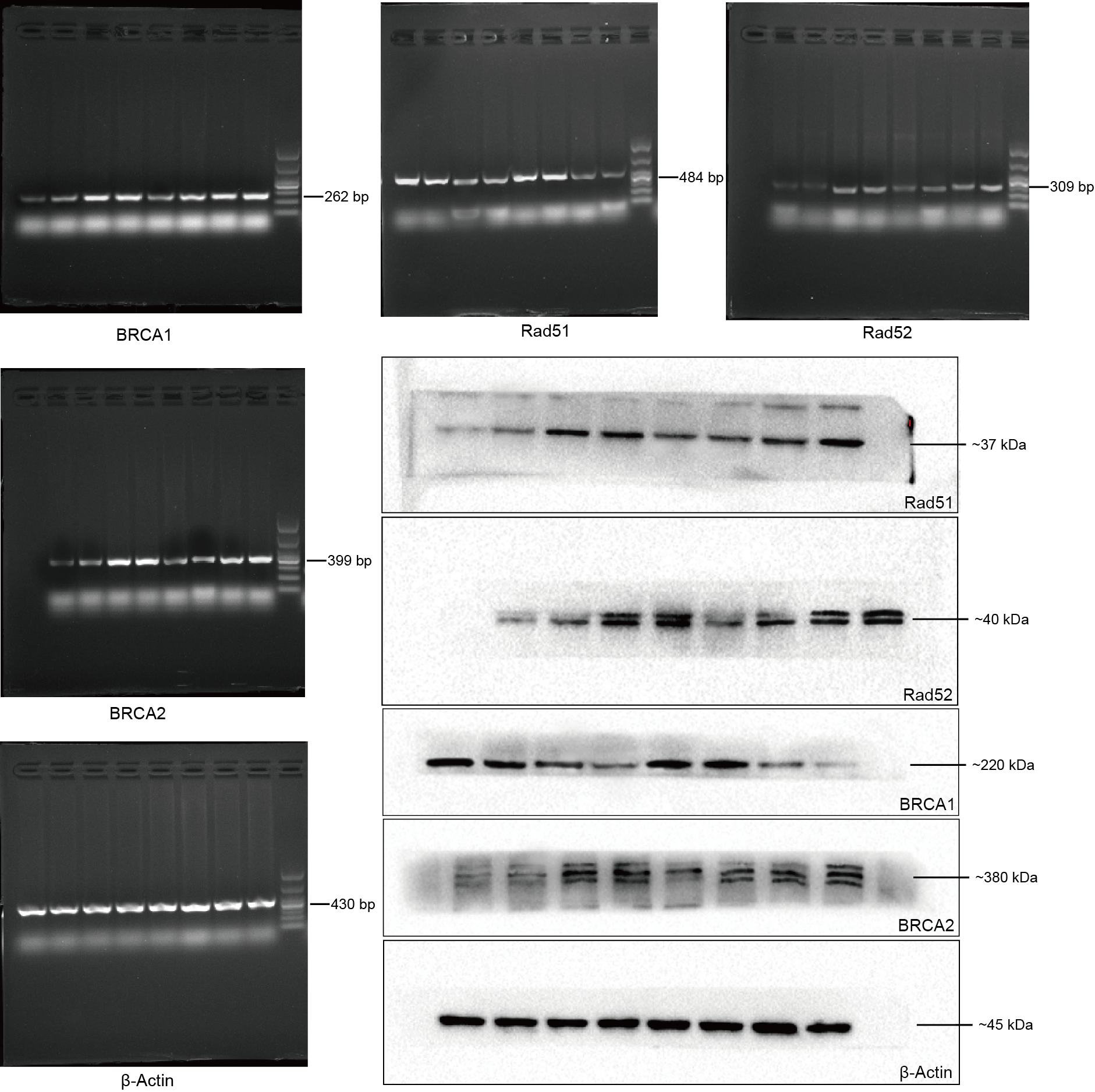
**

**Figure S4. Full length images of Rad51, Rad52, BRCA1, BRCA2 and β-Actin.** The dilution ratios of all primary antibodies are 1:1000. The marker of DNA ladder from top to down are 700 bp, 600 bp, 500 bp, 400 bp, 300 bp, 200 bp and 100 bp, respectively.

**
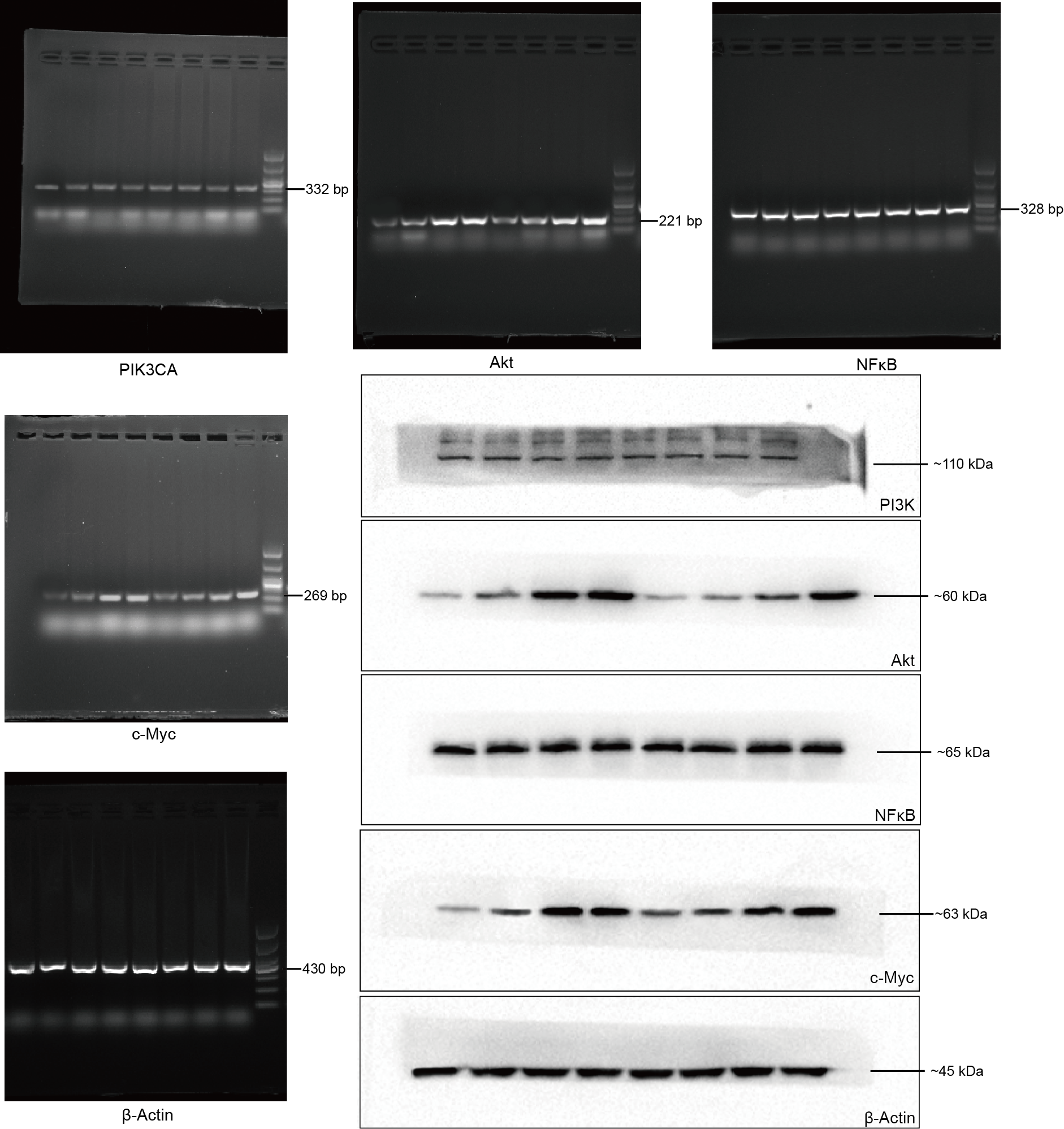
**

**Figure S5. Full length images of PI3K, Akt, NFκB, c-Myc and β-Actin.** The dilution ratios of all primary antibodies are 1:1000. The marker of DNA ladder from top to down are 700 bp, 600 bp, 500 bp, 400 bp, 300 bp, 200 bp and 100 bp, respectively.

**
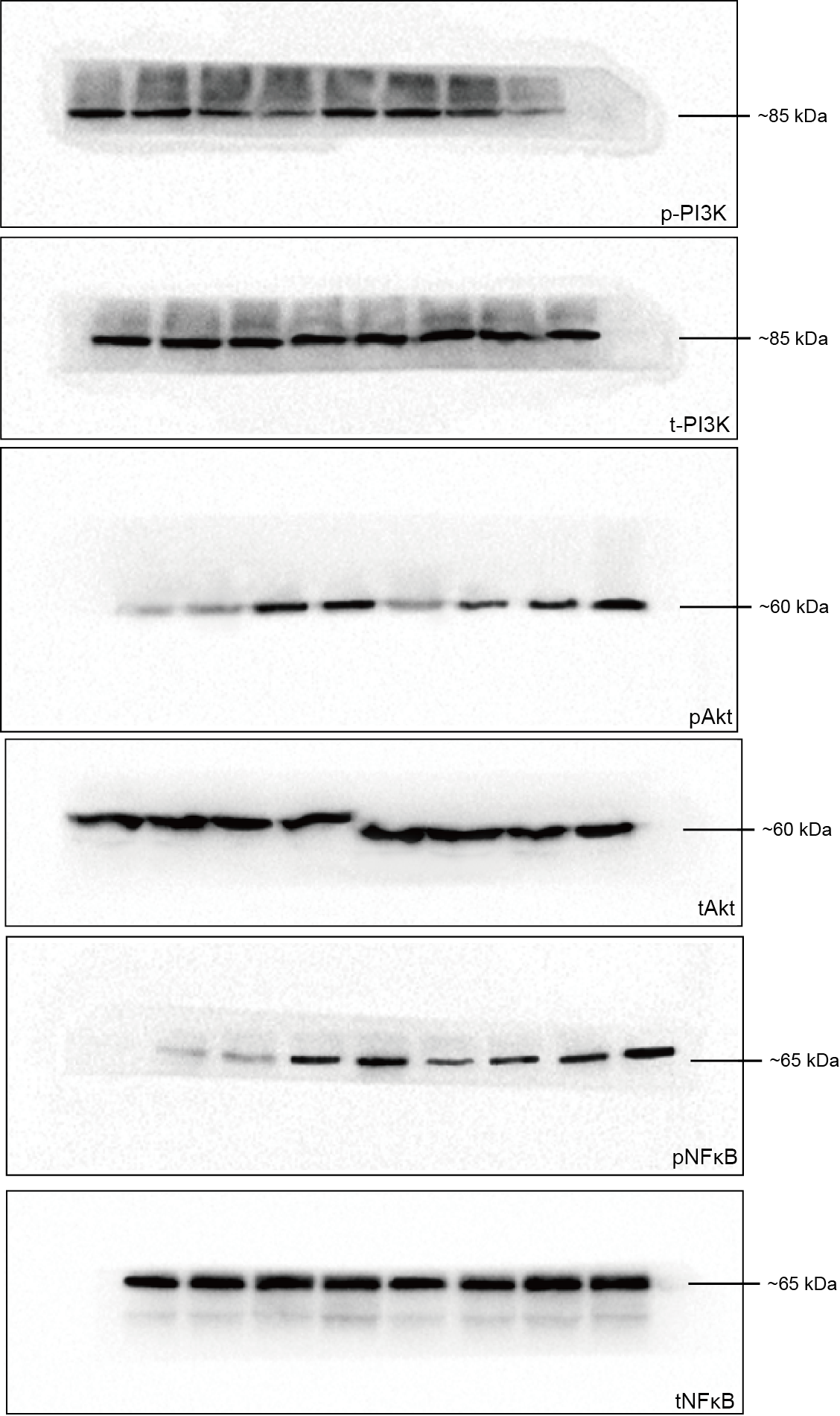
**

**Figure S6. Full length images of c-Myc, pc-Myc, PI3K, pPI3K, NFκB and pNFκB.** The dilution ratios of all primary antibodies are 1:1000.

**
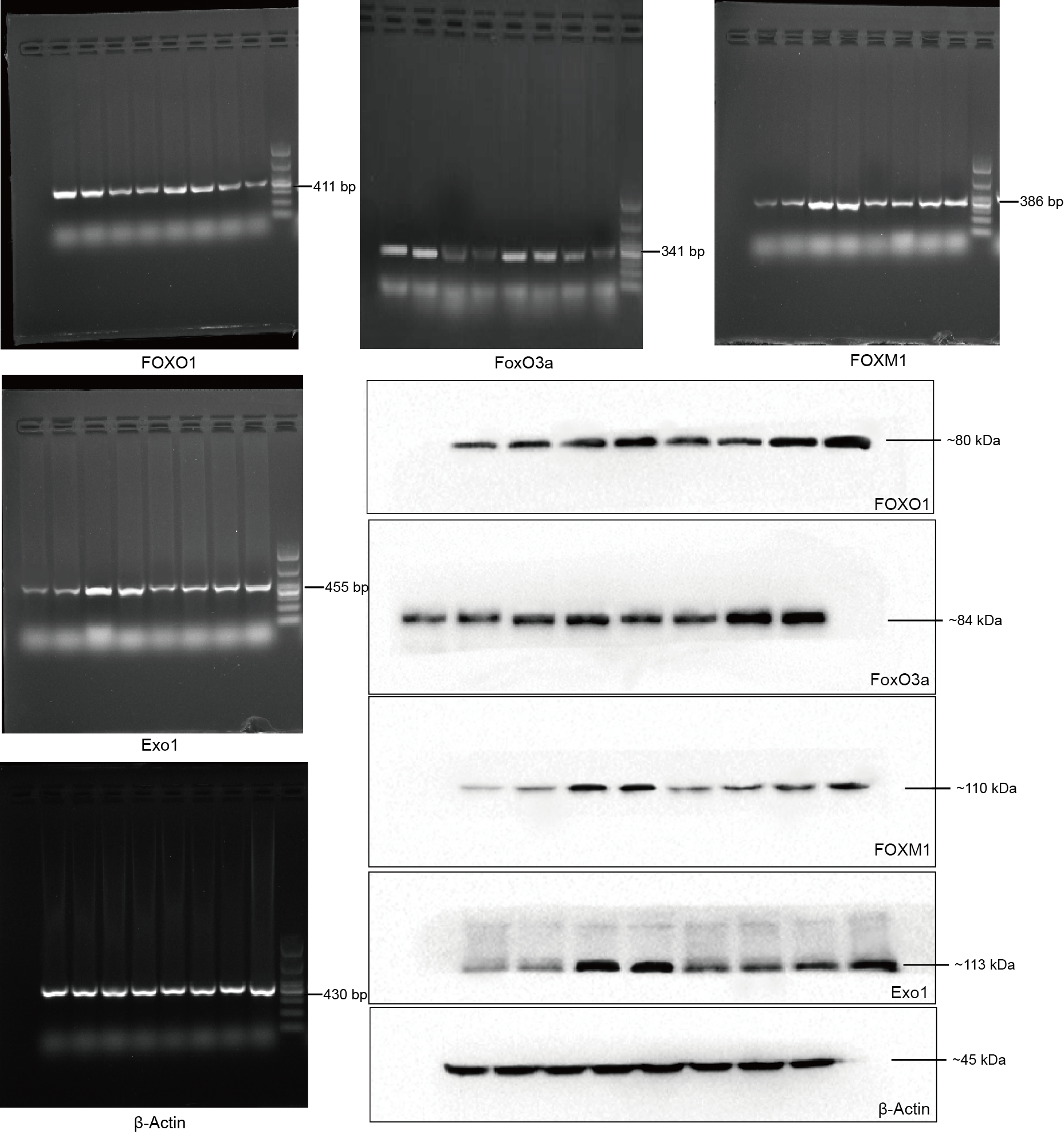
**

**Figure S7. Full length images of FOXO1, FoxO3a, FOXM1, Exo-1 and β-Actin.** The dilution ratios of all primary antibodies are 1:1000. The marker of DNA ladder from top to down are 700 bp, 600 bp, 500 bp, 400 bp, 300 bp, 200 bp and 100 bp, respectively.

**
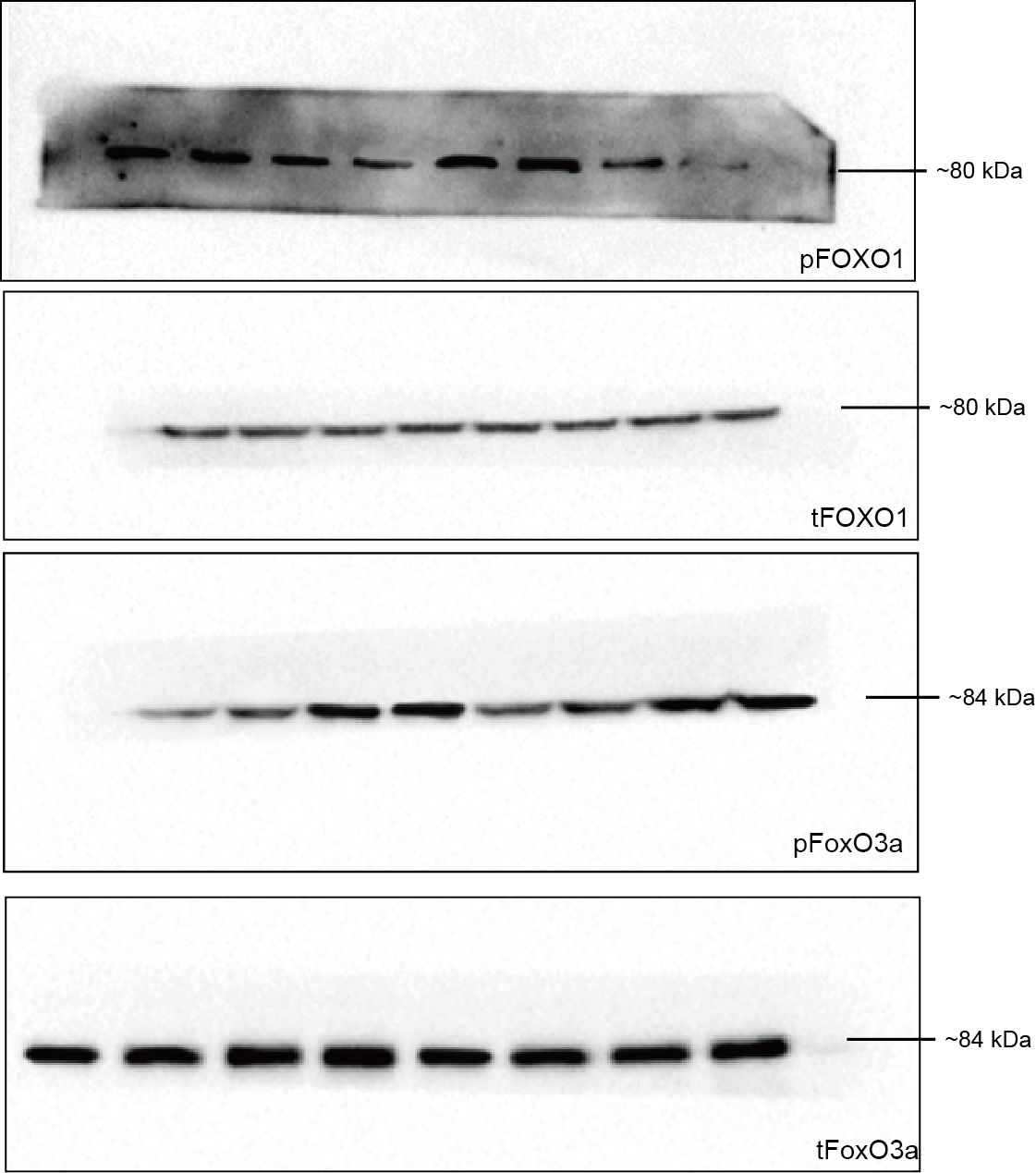
**

**Figure S8. Full length images of pFOXO1, FOXO1, pFoxO3a and FoxO3a.** The dilution ratios of all primary antibodies are 1:1000.

**
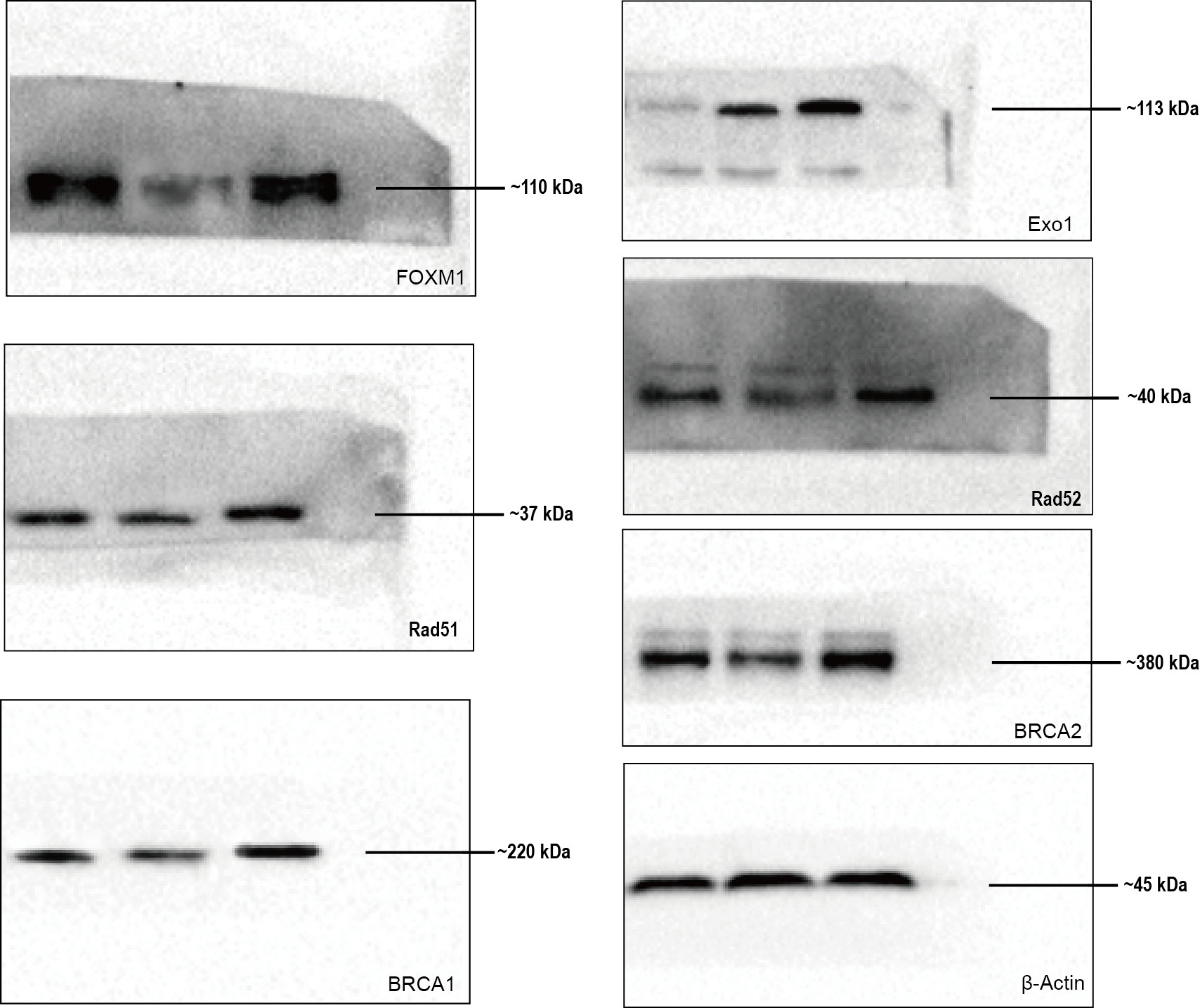
**

**Figure S9. Full length images of FOXM1, Exo-1, BRCA1, BRCA2, Rad51, Rad52 and β-Actin.** The dilution ratios of all primary antibodies are 1:1000.
